# Crossover recombination and synapsis are linked by adjacent regions within the N terminus of the Zip1 synaptonemal complex protein

**DOI:** 10.1371/journal.pgen.1008201

**Published:** 2019-06-20

**Authors:** Karen Voelkel-Meiman, Shun-Yun Cheng, Melanie Parziale, Savannah J. Morehouse, Arden Feil, Owen R. Davies, Arnaud de Muyt, Valérie Borde, Amy J. MacQueen

**Affiliations:** 1 Department of Molecular Biology and Biochemistry, Wesleyan University, Middletown, Connecticut, United States of America; 2 Department of Ophthalmology and Gene Therapy Center, University of Massachusetts Medical School, Worcester, Massachusetts, United States of America; 3 Larner College of Medicine, University of Vermont, Burlington, Vermont, United States of America; 4 University of Rochester School of Medicine & Dentistry, Rochester, New York, United States of America; 5 Institute for Cell and Molecular Biosciences, Newcastle University, Newcastle upon Tyne, United Kingdom; 6 Institut Curie, PSL Research University, CNRS, UMR3244, Paris, France; 7 Université Pierre et Marie Curie (UPMC), Paris, France; National Cancer Institute, UNITED STATES

## Abstract

Accurate chromosome segregation during meiosis relies on the prior establishment of at least one crossover recombination event between homologous chromosomes. Most meiotic recombination intermediates that give rise to interhomolog crossovers are embedded within a hallmark chromosomal structure called the synaptonemal complex (SC), but the mechanisms that coordinate the processes of SC assembly (synapsis) and crossover recombination remain poorly understood. Among known structural components of the budding yeast SC, the Zip1 protein is unique for its independent role in promoting crossover recombination; Zip1 is specifically required for the large subset of crossovers that also rely on the meiosis-specific MutSγ complex. Here we report that adjacent regions within Zip1’s N terminus encompass its crossover and synapsis functions. We previously showed that deletion of Zip1 residues 21–163 abolishes tripartite SC assembly and prevents robust SUMOylation of the SC central element component, Ecm11, but allows excess MutSγ crossover recombination. We find the reciprocal phenotype when Zip1 residues 2–9 or 10–14 are deleted; in these mutants SC assembles and Ecm11 is hyperSUMOylated, but MutSγ crossovers are strongly diminished. Interestingly, Zip1 residues 2–9 or 2–14 are required for the normal localization of Zip3, a putative E3 SUMO ligase and pro-MutSγ crossover factor, to Zip1 polycomplex structures and to recombination initiation sites. By contrast, deletion of Zip1 residues 15–20 does not detectably prevent Zip3’s localization at Zip1 polycomplex and supports some MutSγ crossing over but prevents normal SC assembly and Ecm11 SUMOylation. Our results highlight distinct N terminal regions that are differentially critical for Zip1’s roles in crossing over and SC assembly; we speculate that the adjacency of these regions enables Zip1 to serve as a liaison, facilitating crosstalk between the two processes by bringing crossover recombination and synapsis factors within close proximity of one another.

## Author summary

Reproductive cell formation relies on a nuclear division cycle called meiosis, wherein two homologous sets of chromosomes are reduced to one. At the crux of (and critically required for) meiotic chromosome segregation is a transient association between homologous chromosomes established by a crossover recombination event. Recombination intermediates embed within a ~100 nm wide proteinaceous structure that connects aligned homologous axes, the synaptonemal complex (SC). While genetic data implicate certain SC structural proteins in crossover formation, it is unclear how such coiled-coil, rod-like proteins carry out their recombination function. Our structure-function analysis of the yeast SC transverse filament protein, Zip1, reveals pro-crossover and pro-synapsis functions that are encompassed by adjacent N terminal regions. We also discovered that the pro-crossover region of Zip1 promotes proper localization of pro-crossover factor and putative SUMO ligase, Zip3, to meiotic recombination sites. Zip3 is known to not only promote crossovers but also to influence the post-translational modification of another SC structural component, Ecm11, which is dispensable for crossovers. Our findings raise the possibility that Zip1’s N terminus acts as a liaison to connect pro-crossover factors (like Zip3) to SC assembly proteins (such as Ecm11) in order to coordinate the two landmark meiotic chromosomal processes.

## Introduction

A unique feature of the meiotic cell cycle is how chromosomes are segregated at the first division: Homologous chromosomes (homologs) orient and precisely separate from one another on the meiosis I spindle due to the prior establishment of recombination-based associations between homologs. Interhomolog crossover recombination creates a reciprocal splice between non-sister DNA molecules; in conjunction with sister cohesion, this DNA exchange provides a physical association between replicated homologs that is stable but nevertheless can be released to allow disjunction after the bivalent has acquired a proper orientation on the spindle [1].

Interhomolog crossovers form during meiotic prophase through the homologous recombination-based repair of a large number of programmed DSBs catalyzed by the meiosis-specific, topoisomerase-like protein, Spo11 [2]. For many organisms, the repair pathway that allows a subset of Spo11-mediated DSBs to become interhomolog crossovers involves the formation and processing of Holliday junction intermediates by meiosis-specific proteins [3, 4]. This set of crossover-promoting proteins includes the MutSγ (Msh4-Msh5) and MutLγ(Mlh1-Mlh3) heterodimeric complexes which have homology to the bacterial MutS and MutL protein families, respectively [5–10]. DSB repair processes in meiotic cells also rely on meiosis-specific proteins and pathways to ensure desired outcomes unique to meiosis: for example that crossovers preferentially involve non-sister chromatids of homologous chromosomes (as opposed to involving the sister chromatids that comprise a single chromosome), and that every chromosome pair, no matter how small, receives at least one crossover.

Many of the meiosis-specific factors that function in crossover recombination also function to promote the assembly of a widely-conserved, prominent feature of meiotic prophase chromosomes: the synaptonemal complex (SC) [11]. The SC is a proteinaceous macromolecular structure comprised largely of proteins (called transverse filaments) that feature extensive regions of predicted coiled-coil secondary structure. These rod-like transverse filament proteins assemble in an ordered fashion to create “rungs” connecting aligned chromosome axes along their entire lengths (chromosome axis structures are referred to as lateral elements in the context of the mature SC structure). As demonstrated in multiple organisms by electron and super-resolution microscopy using epitope-specific antibodies, SC transverse filament protein units span the conserved 100 nm width of the SC and orient with their opposing C termini toward lateral element structures [12–17]. In many organisms including budding yeast and mammals, a distinct set of SC structural proteins assemble near the N terminal regions of transverse filaments at the midline of the SC, comprising the “central element” substructure [13, 18, 19].

While SC assembly (synapsis) and recombination are mechanistically independent and separable, crosstalk exists between the two processes. One widely-conserved example of such crosstalk is the reliance of meiotic crossover events on proteins that are also essential for SC assembly. Especially noteworthy is the fact that mutants missing structural building block components of the SC, particularly transverse filament proteins such as the budding yeast Zip1 protein, are typically deficient in MutSγ crossover formation [11]. The reliance of crossover recombination on SC proteins is perhaps unsurprising given that meiotic recombination intermediate-associated complexes fated to become chiasmata (cytological manifestations of the crossover links between homologs) embed directly within the central region of the SC [20–23]. However we note that, at least in budding yeast, not all SC structural components play a role in crossing over and the mature SC structure itself is not a prerequisite for crossover recombination: Budding yeast mutants deficient in the SC central element components Ecm11 or Gmc2, or expressing an *ecm11* allele that prevents Ecm11 SUMOylation, fail to assemble tripartite SC but nevertheless exhibit crossovers, which remain MutSγ-dependent and actually occur in excess compared to wild-type [24]. This finding indicates not only that tripartite SC is dispensable for crossing over, but that SC is associated with an activity that antagonizes interhomolog crossover formation; at least one aspect of the observed anti-crossover activity of the budding yeast SC is likely to be a capacity to inhibit Spo11 DSB formation [25, 26].

Genetic evidence from multiple systems also suggests that meiotic recombination directly influences SC assembly. In organisms including budding yeast and mammals, early steps in homologous recombination are a prerequisite for proper synapsis. In *spo11* mutants, which fail to initiate meiotic recombination, SC assembly does not occur extensively in mammals, or at all in budding yeast [2, 27]. Furthermore, SC assembly in budding yeast is initiated both from centromeres and also from interstitial chromosomal sites that are presumed to be recombination-associated [28, 29]. A subset of proteins that co-localize with MutSγ on budding yeast meiotic chromosomes are required downstream of DSB formation not only for the formation of stable, crossover-designated recombination intermediates but also for robust SC assembly [30, 31]. This group of proteins includes the so-called “Synapsis Initiation Complex” (SIC) factors (Zip2, Zip3, Zip4 and Spo16 [28, 32–34]). In the absence of any one of these proteins, the MutSγ heterodimer, or the SC transverse filament protein Zip1, recombination intermediates fail to form stable joint molecule (Holliday junction) structures [30, 35]. Recent studies indicate that Zip2, Zip4 and Spo16 form a complex and that Zip2 and Spo16 together can bind branched DNA structures, which suggests a structural basis for the role of these proteins in stabilizing recombination intermediates [36, 37]. In the absence of Zip2, Zip4 or Spo16 (and, by default, Zip1), SC assembly is also abolished. The absence of MutSγ or the putative E3 SUMO ligase, Zip3, does not cause a complete absence but rather a diminishment of SC assembly, presumably due to a failure or severe delay in synapsis initiation from non-centromeric chromosomal sites [28, 29]. Finally, SIC protein activity has been found to be required for normal SUMOylation of the SC central element component, Ecm11, which is critical for SC elaboration [38]. Taken together, these data suggest that intermediate events in the budding yeast meiotic recombination process mediate the gradual, stepwise assembly of a recombination intermediate-associated complex that has the capacity to trigger SC elaboration.

Interestingly, even in *C*. *elegans* where SCs assemble in the absence of recombination initiation, MutSγ-associated crossover recombination intermediates locally influence the physical and dynamic properties of the *C*. *elegans* SC, through a Polo-like kinase (PLK-2) signaling mechanism [39]. The observed interdependencies between synapsis and meiotic recombination indicate that the two processes are not only spatially correlated but also functionally intertwined. However, we currently lack a substantial molecular understanding of the how these hallmark meiotic processes intersect.

In budding yeast it is clear that the SC transverse filament protein, Zip1, serves an early role in promoting crossover recombination independent of (and prior to) its structural role in assembling the SC [24, 30]. In this case, a single protein evolved dual functions to promote these distinct but coordinated meiotic prophase processes. The budding yeast Zip1 protein thus provides an opportunity to understand how SC transverse filaments can both promote and coordinate interhomolog recombination and SC assembly. Here we present a phenotypic analysis of mutants carrying a series of non-null *zip1* alleles that encode small in-frame deletions within Zip1’s (putatively unstructured) N terminus; taken together with our previously-published analysis of the *zip1[Δ21–163]* mutant, these *zip1* alleles encompass distinct and nearly reciprocal phenotypes with respect to synapsis and crossover recombination. These separation-of-function mutants reveal critical N-terminal residues that correspond to Zip1’s dual function in regulating crossing over and synapsis, and suggest that these residues may encompass adjacent interaction sites for the pro-crossover factor and putative E3-SUMO ligase, Zip3, and the SUMOylated SC central element component, Ecm11.

## Results

### MutSγ crossovers rely on residues within Zip1’s extreme N terminus

**Fig 1:**
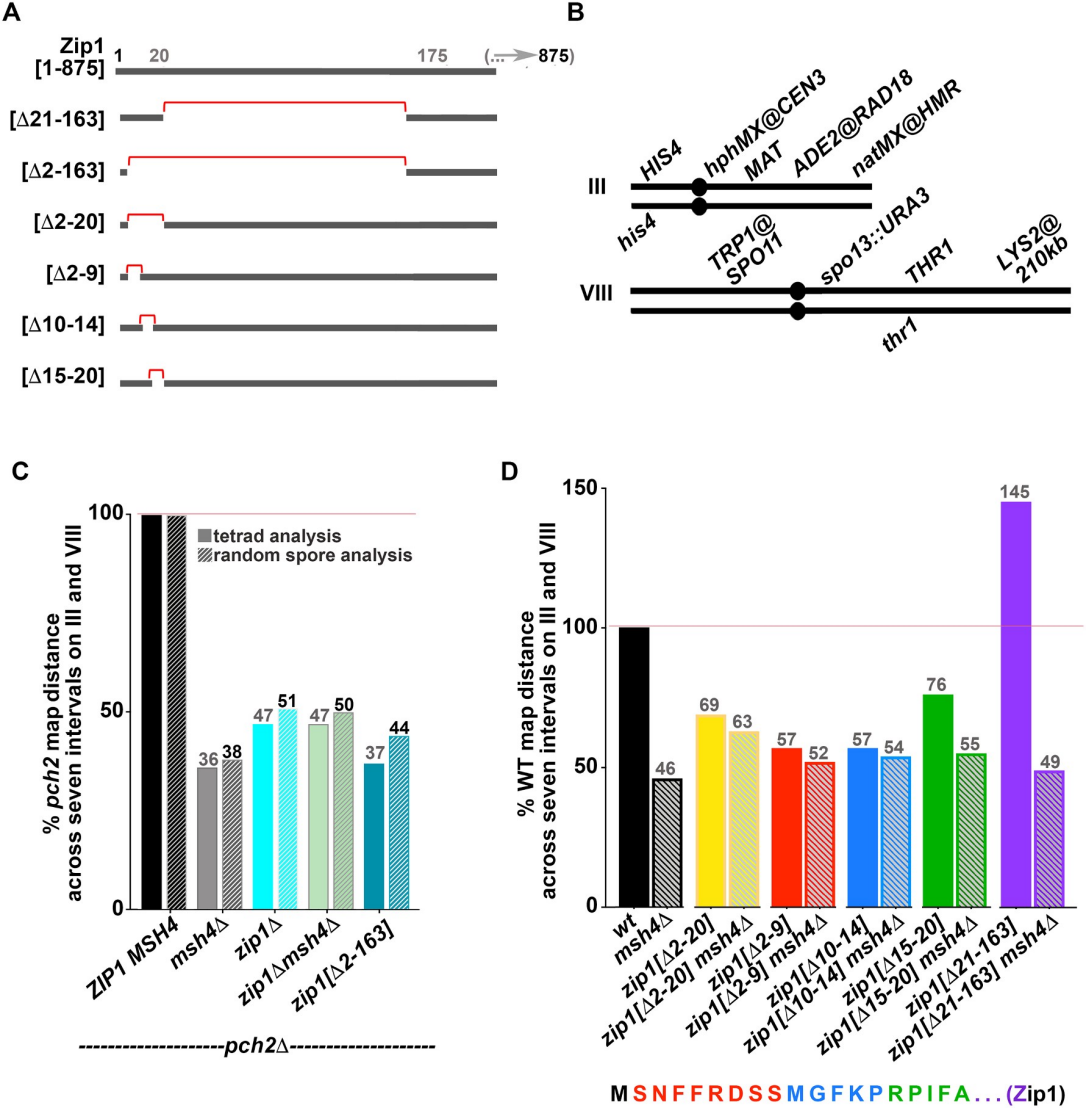
Zip1’s N terminal twenty residues are critical for MutSγ crossing over. **A)** Line illustrations represent Zip1’s N terminal region (approximately residues 1–200); red brackets highlight residues that are deleted in the altered versions of Zip1 analyzed in this study (precise deletion information is indicated at the left of each line). **B)** Cartoons illustrate the seven genetic intervals that were utilized to assess meiotic crossover recombination in this study; strains carried two distinguishable alleles (genetic markers) corresponding to five loci whose position span the length of chromosome III, and four loci whose positions encompass more than half of chromosome VIII. Crossover recombination is measured by examining the frequency of chromatids for which adjacent genetic markers have become unlinked from one another during meiosis; a higher frequency of crossover recombination results in a larger map distance between genetic markers that define an interval. (**C, D**) Bar graphs plot the sum of map distances across all seven intervals in each strain, normalized to the control value. Note that the control for normalization in (**C**) is the *pch2* value, while wild type is the control in (**D**). Random spore analysis was used to calculate the map distances shown in the hatched bars graphed in **(C)**, while tetrad analysis was used to calculate the map distances illustrated by the solid bars in **(C, D)**; see Materials and methods for map distance calculation procedures. Exact values corresponding to % of control map distances are indicated in gray color above each bar in (**C, D**). Map distances for individual intervals are reported in Table 1 (*pch2* strains) and Table 2 (*PCH2* strains). *pch2* strains used in (**C)** are: AM3724 (*ZIP1 MSH4*), AM4025 *(msh4*), AM4023 (*zip1*), AM4026 (*zip1 msh4*), AM3725 (*zip1[Δ2–163])*. Strains used in (**D)** are: K842 (*wt*), K852 (*msh4*), AM3684 (*zip1[Δ2–20])*, K1000 (*zip1[Δ2–20] msh4)*, MP43 (*zip1[Δ2–9])*, MP46 (*zip1[Δ2–9] msh4)*, SYC107 (*zip1[Δ10–14])*, SYC149 *zip1[Δ10–14] msh4*, AF8 (*zip1[Δ15–20])*, K914 (*zip1[Δ15–20] msh4)*, AF6 (*zip1[Δ21–163])*, and SYC151 (*zip1[Δ21–163] msh4)*. Data for wild-type, *msh4*, *zip1[Δ21–163]*, and *zip1[Δ21–163] msh4* strains were reported previously [24]. Zip1’s first twenty amino acid residues are illustrated below the graph in (**D**), with colors corresponding to the residues removed in each of three *zip1* mutant alleles: red = *zip1[Δ2–9]*, blue = *zip1[Δ10–14]*, green = *zip1[Δ15–20]*.

The primary amino acid sequence of Zip1 suggests that the region encompassing residues ~175–748 of the 875 residue protein has the capacity to assemble an extended coiled-coil structure, while the flanking N- and C-terminal regions are likely unstructured. Mirror-image Zip1 units assemble in a head-to-head fashion to span the ~100 nm width of the budding yeast SC central region [12, 13]; Zip1’s C termini orient toward aligned chromosome axes (lateral elements) while its N termini orient toward the central element substructure (comprised of–at least–the SUMO, Ecm11, and Gmc2 proteins) at the midline of the budding yeast SC. We previously reported that the non-null *zip1[Δ21–163]* mutant phenocopies SC central element-deficient *ecm11* and *gmc2* null mutants. In the *zip1[Δ21–163]*, *ecm11* (null) or *gmc2* (null) mutant, tripartite SC assembly fails but MutSγ-mediated crossover recombination events occur in excess [24]. In order to identify residues within the Zip1 and Zip1[Δ21–163] proteins that are critical for Zip1’s crossover activity, we created and analyzed additional non-null *zip1* alleles (Fig 1A). We found that alleles encoding disruptions in Zip1’s first twenty residues severely diminish Zip1’s capacity to promote MutSγ crossovers (Fig 1B, 1C and 1D).

Unlike *zip1[Δ21–163]* mutants but similar to a *zip1* null (corresponding to a complete deletion of the *ZIP1* ORF) in our BR1919 strain background, *zip1[Δ2–163]* meiotic cells exhibit a severely diminished capacity to form spores (1% in *zip1[Δ2–163]* vs 5% in *zip1* vs 57% in the wild-type strain, n > 1000; S1 Table). The meiotic checkpoint that prevents *zip1* null meiotic cells from producing spores relies on the AAA+ ATPase protein, Pch2 [40, 41], thus we removed *PCH2* activity from *zip1[Δ2–163]* strains in order to evaluate the spore viability and meiotic crossover recombination phenotypes associated with this allele. As expected, the diminished spore formation phenotype of *zip1[Δ2–163]* is partially bypassed by removal of *PCH2* (28%; S1 Table). We found that meiotic products from *pch2 zip1[Δ2–163]* strains were only slightly more viable than those from *pch2 zip1* strains (58% in *zip1[Δ2–163]* vs. 39% in *zip1*; S1 Table), raising the possibility that the pro-crossover activity of Zip1 (and of Zip1[Δ21–163]) relies on Zip1 residues 2–20. Indeed, consistent with previously published data implicating Zip1 in the formation of MutSγ crossovers, tetrad or random spore analysis to deduce meiotic crossover recombination frequencies within seven distinct intervals (spanning most of chromosome III and a substantial length of chromosome VIII; Fig 1B) revealed that *pch2 msh4*, *pch2 zip1*, and *pch2 zip1[Δ2–163]* meiotic cells exhibit similarly diminished interhomolog crossing over relative to the *pch2* single mutant (Fig 1C, Table 1). These data in conjunction with our prior phenotypic analysis of *zip1[Δ21–163]* and *zip1[Δ21–163] msh4* [24] indicates a critical role for the first twenty residues of Zip1 in promoting MutSγ crossovers.

**Table 1:** Genetic map distances in mutant strains; pch2 background.

		TETRAD ANALYSIS									RANDOM SPORE					
GENOTYPE (STRAIN)	INTERVAL (CHROMOSOME)	PD	TT	NPD	TOTAL	cM (± SE)	% *pch2*	cM by chrm	% *pch2* by chrm	NPDobs/NPDexp (± SE)	# CO spores	# viable spores	% recombinant	% *pch2*	cM by chrm	% *pch2* by chrm
*pch2Δ* (AM3724)	*HIS4-CEN3* (III)	254	255	12	521	**31.4 (2.1)**	**100**	137.8 (III)	**100**	0.47 (0.14)	726	2714	**26.8**	**100**	113.4 (III)	**100**
	*CEN3-MAT* (III)	265	253	13	531	**31.2 (2.1)**	**100**			0.54 (0.16)	713		**26.3**	**100**		
	*MAT-RAD18* (III)	168	317	29	514	**47.8 (2.9)**	**100**			0.55 (0.12)	975		**35.9**	**100**		
	*RAD18-HMR* (III)	282	221	10	513	**27.4 (2.0)**	**100**			0.57 (0.19)	663		**24.4**	**100**		
	*SPO11-SPO13* (VIII)	180	310	38	528	**51.0 (3.2)**	**100**	125.6 (VIII)	**100**	0.84 (0.16)	988		**36.4**	**100**	92.6(VIII)	**100**
	*SPO13-THR1* (VIII)	362	146	3	511	**16.1 (1.4)**	**100**			0.46 (0.27)	460		**16.9**	**100**		
	*THR1-LYS2* (VIII)	142	301	45	488	**58.5 (3.6)**	**100**			0.90 (0.17)	1066		**39.3**	**100**		
*pch2Δ msh4Δ* (AM4025)	*HIS4-CEN3* (III)	161	34	2	197	**11.7 (2.5)**	**37**	50.7 (III)	**37**	2.40 (1.73)	142	1684	**8.4**	**31**	38.2 (III)	**34**
	*CEN3-MAT* (III)	179	22	1	202	**6.9 (1.8)**	**22**			3.09 (3.11)	87		**5.2**	**20**		
	*MAT-RAD18* (III)	139	60	2	201	**17.9 (2.6)**	**37**			0.70 (0.50)	235		**14.0**	**39**		
	*RAD18-HMR* (III)	149	51	1	201	**14.2 (2.1)**	**52**			0.51 (0.51)	180		**10.7**	**44**		
	*SPO11-SPO13* (VIII)	162	35	0	197	**8.9 (1.4)**	**17**	43.9 (VIII)	**35**	n.d.	163		**9.7**	**18**	40.0 (VIII)	**43**
	*SPO13-THR1* (VIII)	165	23	0	188	**6.1 (1.2)**	**38**			n.d.	119		**7.1**	**42**		
	*THR1-LYS2* (VIII)	99	84	4	187	**28.9 (3.4)**	**49**			0.56 (0.29)	392		**23.3**	**59**		
*pch2Δ zip1Δ* (AM4023)	*HIS4-CEN3* (III)	78	16	0	94	**8.5 (1.9)**	**27**	62.6 (III)	**45**	n.d.	124	1208	**10.3**	**38**	46.7 (III)	**41**
	*CEN3-MAT* (III)	69	27	1	97	**17.0 (3.7)**	**54**			0.85 (0.87)	134		**11.1**	**42**		
	*MAT-RAD18* (III)	62	28	2	92	**21.7 (4.9)**	**45**			1.46 (1.08)	180		**14.9**	**42**		
	*RAD18-HMR* (III)	75	17	2	94	**15.4 (4.8)**	**56**			4.55 (3.33)	126		**10.4**	**43**		
	*SPO11-SPO13* (VIII)	56	40	1	97	**23.7 (3.8)**	**46**	61.4 (VIII)	**49**	0.33 (0.34)	267		**22.1**	**61**	58.9 (VIII)	**64**
	*SPO13-THR1* (VIII)	74	14	2	90	**14.4 (4.9)**	**89**			6.56 (1.80)	122		**10.1**	**60**		
	*THR1-LYS2* (VIII)	53	36	1	90	**23.3 (4.0)**	**40**			0.39 (0.40)	322		**26.7**	**68**		
*pch2Δ zip1[Δ2–163]* (AM3725)	*HIS4-CEN3* (III)	265	50	0	315	**7.9 (1.0)**	**25**	48.0 (III)	**35**	n.d.	225	2494	**9.0**	**34**	44.3 (III)	**39**
	*CEN3-MAT* (III)	263	57	0	320	**8.9 (1.1)**	**26**			n.d.	225		**9.0**	**34**		
	*MAT-RAD18* (III)	203	104	5	312	**21.5 (2.4)**	**45**			0.87 (0.40)	409		**16.4**	**46**		
	*RAD18-HMR* (III)	258	55	1	314	**9.7 (1.4)**	**35**			0.73 (0.73)	246		**9.9**	**41**		
	*SPO11-SPO13* (VIII)	226	82	2	310	**15.2 (1.8)**	**30**	50.5 (VIII)	**40**	0.60 (0.43)	39		**13.6**	**37**	46.0 (VIII)	**50**
	*SPO13-THR1* (VIII)	253	31	0	284	**5.5 (0.9)**	**34**			n.d.	201		**8.1**	**48**		
	*THR1-LYS2* (VIII)	155	121	8	284	**29.8 (3.1)**	**51**			0.84 (0.31)	606		**24.3**	**62**		
*pch2Δ zip1Δ msh4Δ* (AM4026)	*HIS4-CEN3* (III)	21	2	0	23	**4.3 (2.9)**	**14**	60.2 (III)	**44**	n.d.	56	529	**10.6**	**40**	46.7 (III)	**41**
	*CEN3-MAT* (III)	17	7	0	24	**14.6 (4.6)**	**47**			n.d.	63		**11.9**	**45**		
	*MAT-RAD18* (III)	14	9	1	24	**31.3 (12.4)**	**65**			1.71 (1.84)	77		**14.6**	**41**		
	*RAD18-HMR* (III)	20	5	0	25	**10.0 (4.0)**	**36**			n.d.	51		**9.6**	**39**		
	*SPO11-SPO13* (VIII)	11	10	0	21	**23.8 (5.5)**	**47**	62.5 (VIII)	**50**	n.d.	154		**29.1**	**80**	56.0 (VIII)	**60**
	*SPO13-THR1* (VIII)	20	2	0	22	**4.6 (3.1)**	**29**			n.d.	47		**8.9**	**53**		
	*THR1-LYS2* (VIII)	12	9	1	22	**34.1 (13.4)**	**58**			1.50 (1.64)	95		**18.0**	**46**		

We next assessed the sporulation and interhomolog crossover recombination phenotypes of *zip1* alleles that encode proteins with smaller internal deletions of Zip1’s N terminus (Fig 1A). We found that, in contrast to *zip1[Δ2–163]*, strains expressing *zip1[Δ2–9]*, *zip1[Δ10–14]*, and *zip1[Δ15–20]* sporulate as efficiently as *zip1[Δ21–163]* and wild type BR strains (57%, 54%, and 51%, respectively; S1 Table). *zip1[Δ2–20]* gave an intermediate sporulation efficiency at 20%. These data indicate that Zip1’s residues 2–20 and 21–163 contribute redundantly to the mechanism(s) that normally prevent a *PCH2*-mediated checkpoint block to meiotic progression.

Despite their near normal sporulation efficiency and high spore viability (S1 Table), *zip1[Δ2–20]*, *zip1[Δ2–9]*, *zip1[Δ10–14]*, and *zip1[Δ15–20]* mutants exhibit deficiencies in MutSγ-mediated crossover recombination. Removal of the MutSγ complex protein, Msh4, reduces detectable crossovers in chromosome III and VIII genetic intervals to about 50% of wild-type levels (Fig 1D, Table 2). By contrast to *zip1[Δ21–163]* single mutants, which exhibit elevated crossovers [24], *zip1[Δ2–20]*, *zip1[Δ2–9]*, *zip1[Δ10–14]*, and *zip1[Δ15–20]* mutants exhibit 69%, 57%, 57% and 76% of the corresponding wild-type crossover level, respectively; Fig 1D, Table 2). Importantly, removal of *MSH4* from *zip1[Δ2–20]*, *zip1[Δ2–9]*, and *zip1[Δ10–14]* strains resulted in little change in the crossover phenotype relative to their *zip1* single mutant allele counterparts, (*zip1[Δ2–20] msh4*, *zip1[Δ2–9] msh4*, and *zip1[Δ10–14] msh4* double mutants exhibit 63%, 52%, and 54% of wild-type crossovers, respectively). Because removal of Msh4 does not cause a substantial further reduction in crossovers, we conclude that few, if any, MutSγ-mediated crossovers form in *zip1[Δ2–20]*, *zip1[Δ2–9]*, and *zip1[Δ10–14]* mutant strains. By contrast, removal of *MSH4* did reduce the crossover level of the *zip1[Δ15–20]* mutant (76% in *zip1[Δ15–20]* versus 55% in *zip1[Δ15–20] msh4*; Fig 1C and Table 2), suggesting that the Zip1[Δ15–20] protein may support an intermediate level of MutSγ-mediated crossing over. Taken together, the interhomolog crossover recombination phenotypes of *zip1[Δ2–9]*, *zip1[Δ10–14]*, and *zip1[Δ15–20]* mutants indicate that Zip1’s first twenty residues are critical for its meiotic crossover promoting activity.

**Table 2:** Genetic map distances in mutant strains; PCH2 background.

GENOTYPE (STRAIN)	INTERVAL (CHROMOSOME)	PD	TT	NPD	TOTAL	cM (± SE)	%WT	cM by chrm	%WT by chrm	NPDobs/NPDexp (± SE)
WT* (K842)	*HIS4-CEN3* (III)	344	325	6	675	**26.7 (1.4)**	**100**	106.0 (III)	**100**	0.19 (0.08)
	*CEN3-MAT* (III)	427	250	4	681	**20.1 (1.2)**	**100**			0.25 (0.13)
	*MAT-RAD18* (III)	255	405	14	674	**36.3 (1.8)**	**100**			0.22 (0.06)
	*RAD18-HMR* (III)	395	273	6	674	**22.9 (1.4)**	**100**			0.30 (0.13)
	*SPO11-SPO13* (VIII)	251	401	21	673	**39.2 (2.0)**	**100**	76.3 (VIII)	**100**	0.35 (0.08)
	*SPO13-THR1* (VIII)	565	94	1	660	**7.6 (0.8)**	**100**			0.54 (0.54)
	*THR1-LYS2* (VIII)	296	361	5	662	**29.5 (1.3)**	**100**			0.11 (0.05)
msh4Δ* (K852)	*HIS4-CEN3* (III)	373	96	1	470	**10.9 (1.1)**	**41**	53.3 (III)	**50**	0.35 (0.35)
	*CEN3-MAT* (III)	424	50	1	475	**5.9 (0.9)**	**29**			1.41 (1.42)
	*MAT-RAD18* (III)	275	183	7	465	**24.2 (1.9)**	**67**			0.55 (0.21)
	*RAD18-HMR* (III)	351	115	0	466	**12.3 (1.0)**	**54**			n.d
	*SPO11-SPO13* (VIII)	365	88	3	456	**11.6 (1.4)**	**30**	30.2 (VIII)	**40**	1.22 (0.71)
	*SPO13-THR1* (VIII)	423	27	0	450	**3.0 (0.6)**	**39**			n.d
	*THR1-LYS2* (VIII)	320	129	2	451	**15.6 (1.4)**	**53**			0.34 (0.25)
*zip1[Δ2–20]* (AM3684)	*HIS4-CEN3* (III)	463	107	3	573	**10.9 (1.2)**	**41**	71.3 (III)	**67**	1.04 (0.61)
	*CEN3-MAT* (III)	453	123	2	578	**11.7 (1.1)**	**58**			0.52 (0.37)
	*MAT-RAD18* (III)	315	204	15	534	**27.5 (2.3)**	**76**			1.10 (0.30)
	*RAD18-HMR* (III)	346	197	6	549	**21.2 (1.6)**	**93**			0.50 (0.21)
	*SPO11-SPO13* (VIII)	394	143	5	542	**16.0 (1.5)**	**41**	54.7 (VIII)	**72**	0.86 (0.39)
	*SPO13-THR1* (VIII)	390	88	2	480	**10.4 (1.2)**	**137**			0.87 (0.62)
	*THR1-LYS2* (VIII)	262	203	11	476	**28.3 (2.2)**	**96**			0.69 (0.22)
*zip1[Δ2–20] msh4* (K1000)	*HIS4-CEN3* (III)	365	58	3	426	**8.9 (1.5)**	**33**	60.9 (III)	**57**	2.75 (1.61)
	*CEN3-MAT* (III)	336	97	3	436	**13.2 (1.5)**	**66**			0.94 (0.55)
	*MAT-RAD18* (III)	271	133	10	414	**23.3 (2.4)**	**64**			1.44 (0.47)
	*RAD18-HMR* (III)	297	117	2	416	**15.5 (1.5)**	**68**			0.39 (0.28)
	*SPO11-SPO13* (VIII)	280	104	5	389	**17.2 (2.0)**	**44**	54.5 (VIII)	**71**	1.16 (0.53)
	*SPO13-THR1* (VIII)	299	68	0	367	**9.3 (1.0)**	**122**			n.d.
	*THR1-LYS2* (VIII)	194	160	7	361	**28.0 (2.4)**	**95**			0.52 (0.21)
*zip1[Δ2–9]* (MP43)	*HIS4-CEN3* (III)	428	149	2	579	**13.9 (1.2)**	**52**	72.3 (III)	**68**	0.34 (0.24)
	*CEN3-MAT* (III)	441	140	1	582	**12.5 (1.0)**	**62**			0.20 (0.20)
	*MAT-RAD18* (III)	331	241	8	580	**24.9 (1.7)**	**69**			0.44 (0.16)
	*RAD18-HMR* (III)	361	222	4	587	**21.0 (1.4)**	**92**			0.27 (0.14)
	*SPO11-SPO13* (VIII)	414	156	2	572	**14.7 (1.2)**	**38**	33.7 (VIII)	**44**	0.30 (0.22)
	*SPO13-THR1* (VIII)	526	38	0	564	**3.4 (0.5)**	**45**			n.d.
	*THR1-LYS2* (VIII)	400	165	2	567	**15.6 (1.2)**	**53**			0.26 (0.19)
*zip1[Δ2–9] msh4Δ* (MP46)	*HIS4-CEN3* (III)	389	106	4	499	**13.0 (1.5)**	**49**	63.4 (III)	**60**	1.21(0.61)
	*CEN3-MAT* (III)	396	106	0	502	**10.6 (0.9)**	**53**			n.d.
	*MAT-RAD18* (III)	299	185	9	493	**24.2(2.0)**	**67**			0.75(0.26)
	*RAD18-HMR* (III)	354	136	3	493	**15.6 (1.4)**	**68**			0.51 (0.30)
	*SPO11-SPO13* (VIII)	364	117	3	484	**14.0 (1.4)**	**36**	30.5 (VIII)	**40**	0.70 (0.41)
	*SPO13-THR1* (VIII)	452	24	0	476	**2.5 (0.5)**	**33**			n.d.
	*THR1-LYS2* (VIII)	361	109	4	474	**14.0 (1.6)**	**47**			1.70 (0.54)
*zip1[Δ10–14]* (SYC107)	*HIS4-CEN3* (III)	411	139	5	555	**15.2 (1.5)**	**57**	68.5 (III)	**65**	0.94 (0.43)
	*CEN3-MAT* (III)	443	119	5	567	**13.1 (1.4)**	**65**			1.37 (0.62)
	*MAT-RAD18* (III)	336	219	5	560	**22.2 (1.5)**	**61**			0.33 (0.15)
	*RAD18-HMR* (III)	365	196	1	562	**18.0 (1.1)**	**79**			n.d.
	*SPO11-SPO13* (VIII)	399	149	2	550	**14.6 (1.2)**	**37**		**37**	0.32 (0.23)
*zip1[Δ10–14] msh4Δ* (SYC149)	*HIS4-CEN3* (III)	267	78	4	349	**14.6 (2.0)**	**55**	61.6 (III)	**58**	1.55 (0.79)
	*CEN3-MAT* (III)	286	65	4	355	**12.5 (1.9)**	**62**			2.35 (1.19)
	*MAT-RAD18* (III)	240	103	4	347	**18.3 (2.0)**	**50**			0.82 (0.42)
	*RAD18-HMR* (III)	253	96	3	352	**16.2 (1.8)**	**71**			0.74 (0.43)
	*SPO11-SPO13* (VIII)	247	95	3	345	**16.4 (1.9)**	**42**		**42**	0.74 (0.43)
*zip1[Δ15–20]* (AF8)	*HIS4-CEN3* (III)	409	139	4	552	**14.8 (1.4)**	**55**	84.3 (III)	**80**	0.75 (0.38)
	*CEN3-MAT* (III)	388	172	7	567	**18.9 (1.6)**	**94**			0.84 (0.32)
	*MAT-RAD18* (III)	303	232	14	549	**28.8 (2.1)**	**79**			0.78 (0.22)
	*RAD18-HMR* (III)	348	199	7	554	**21.8 (1.7)**	**95**			0.58 (0.22)
	*SPO11-SPO13* (VIII)	379	168	6	553	**18.4 (1.6)**	**47**	54.9 (VIII)	**72**	0.73 (0.31)
	*SPO13-THR1* (VIII)	433	89	0	522	**8.5 (0.8)**	**112**			n.d.
	*THR1-LYS2* (VIII)	275	238	9	522	**28.0 (1.9)**	**95**			0.43 (0.15)
*zip1[Δ15–20] msh4Δ* (K914)	*HIS4-CEN3* (III)	435	108	0	543	**9.9 (0.9)**	**37**	63.7 (III)	**60**	n.d.
	*CEN3-MAT* (III)	446	104	2	552	**10.5 (1.1)**	**52**			0.71 (0.50)
	*MAT-RAD18* (III)	334	185	13	532	**24.7 (2.2)**	**68**			1.20 (0.35)
	*RAD18-HMR* (III)	358	176	4	538	**18.6 (1.5)**	**81**			0.42 (0.21)
	*SPO11-SPO13* (VIII)	380	134	6	520	**16.4 (1.7)**	**42**		**42**	1.14 (0.47)
*zip1[Δ21–163]* (AF6)	*HIS4-CEN3* (III)	263	310	10	583	**31.7 (1.8)**	**119**	139.9 (III)	**132**	0.28 (0.09)
	*CEN3-MAT* (III)	241	329	13	583	**34.9 (1.9)**	**174**			0.30 (0.09)
	*MAT-RAD18* (III)	207	326	17	550	**38.9 (2.2)**	**107**			0.35 (0.09)
	*RAD18-HMR* (III)	230	320	11	561	**34.4 (1.9)**	**150**			0.25 (0.08)
	*SPO11-SPO13* (VIII)	182	331	44	557	**53.4 (3.2)**	**136**	124.7 (VIII)	**163**	0.89 (0.16)
	*SPO13-THR1* (VIII)	323	194	3	520	**20.4 (1.4)**	**268**			0.24(0.14)
	*THR1-LYS2* (VIII)	161	329	34	524	**50.9 (3.0)**	**173**			0.58 (0.12)
zip1[Δ21–163] msh4Δ* (SYC151)	*HIS4-CEN3* (III)	481	116	2	599	**10.7 (1.1)**	**40**	57.8 (III)	**55**	0.62 (0.44)
	*CEN3-MAT* (III)	496	109	2	607	**10.0 (1.0)**	**50**			0.73 (0.52)
	*MAT-RAD18* (III)	407	185	6	598	**18.5 (1.5)**	**51**			0.65 (0.27)
	*RAD18-HMR* (III)	397	199	4	600	**18.6 (1.3)**	**81**			0.37 (0.19)
	*SPO11-SPO13* (VIII)	437	138	2	577	**13.0 (1.1)**	**33**		**33**	0.40 (0.29)

* Data from these strains was previously published (Voelkel-Meiman 2016)

We note that each *msh4* strain bearing one of these four non-null *zip1* alleles exhibits a slightly higher crossover frequency than the *msh4* single mutant. This phenomenon is particularly dramatic for the *zip1[2–20] msh4* strain, which exhibits 63% of wild type crossover frequency whereas the *msh4* single mutant exhibits 46% of wild-type crossovers (Fig 1D, Table 2). The curious result that crossovers are elevated in *msh4* mutants when Zip1’s N terminus is altered (but not when Zip1 is absent altogether; Fig 1C), raises the possibility that Zip1’s N terminal residues might normally constrain the processing of some interhomolog recombination intermediates in a manner that ensures they are MutSγ-dependent.

### Removal of residues 2–9 or 10–14 results in a novel separation-of-function Zip1 protein that is crossover-deficient but synapsis-proficient

Residues 21–163 within Zip1’s N terminal unstructured region are dispensable for Zip1’s function in crossover recombination but essential for tripartite SC assembly [24]. We investigated whether residues 2–20 are critical for the formation of mature SC by asking whether coincident linear structures of the SC transverse filament protein (Zip1) and the SC central element protein Ecm11 assemble on surface-spread meiotic prophase nuclei from strains carrying wild-type or a mutant *zip1* allele and missing the Ndt80 transcription factor. Ndt80 is required for progression beyond a mid-meiotic prophase stage when full-length SCs are normally assembled [42], thus the *ndt80* null background allows us to maintain cells in sporulation medium for prolonged periods in order to assess the overall capacity of a strain to assemble SC. We utilized a polyclonal antibody targeted against Zip1’s C terminal 264 residues [43] together with an antibody against the MYC epitope tag that is fused to the C terminus of one copy of the *ECM11* gene in these strains.

As expected based on the SC-deficient phenotype of the *zip1[Δ21–163]* mutant, meiotic prophase nuclei from the *zip1[Δ2–163]* mutant strain fail to exhibit extensive Zip1 or Ecm11-MYC coincident linear structures on meiotic chromosomes. At 24 hours after placement into sporulation medium, when ~85% of *ZIP1 ndt80* strains in our BR genetic background exhibit nearly full synapsis [44], *zip1[Δ2–163]* mutants instead display Zip1 or Ecm11-MYC foci of varying sizes, sometimes accompanied by a large “polycomplex” aggregate of these SC central region proteins (S1 Fig). Interestingly, *ndt80* meiotic cells expressing *zip1[Δ2–20]* also fail to exhibit any detectable SC formation, even after 24 hours in sporulation medium (S1 Fig). These data in conjunction with the deficient SC assembly phenotype of *zip1[Δ21–163]* [24] indicate that residues within both the 2–20 and 21–163 regions of Zip1 are required for Zip1’s capacity to assemble SC.

**Fig 2:**
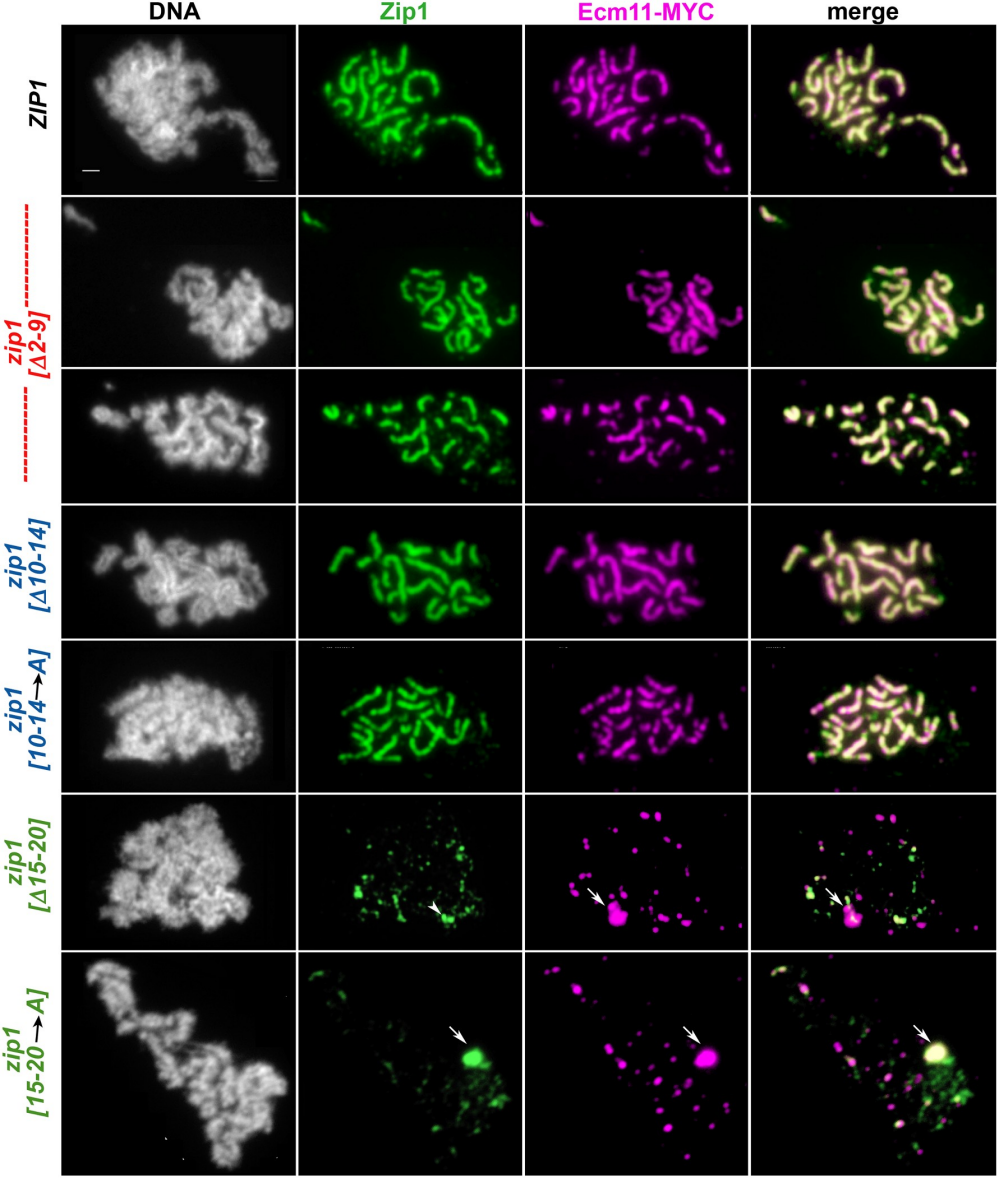
SC assembly requires Zip1 residues 15–20 but not residues 2–14. Panels show representative surface-spread mid-meiotic prophase nuclei from wild-type (top row), and various *zip1* internal deletion or residue-substitution alleles (genotypes indicated at left; strains are K320 (*ZIP1*), AM4064 (*zip1[Δ2–9])*, AM4194 (*zip1[Δ10–14])*, AM4069 (*zip1[10–14→A])*, SYC79 (*zip1[Δ15–20])*, and K981 (*zip1[Δ15–20→A]*). Note that strains carrying a short internal deletion of Zip1 residues 10–14 or 15–20 exhibit a similar phenotype, respectively, as strains in which Zip1 residues 10–14 or 15–20 are replaced with alanine. All strains carry an *ndt80* null allele, which allows meiotic cultures to accumulate at mid-late prophase stages when full-length SCs are normally present. Mid-meiotic prophase chromosomes are stained with DAPI to label DNA (white), anti-Zip1 (green), and anti-MYC to label Ecm11 (magenta). The merge between Zip1 and Ecm11 channels is shown in the final column. Quantitation of the number and cumulative length of SC linear assemblies in wild type as well as each internal deletion *zip1* mutant strain is given in Fig 4. Arrows point to polycomplex structures. Arrowhead indicates a large focus or pair of foci that measures at 0.7 μm and thus may have been included in the assessment of “linear” SC structures (see Fig 4). Scale bar, 1 μm.

However, we found that not all residues within Zip1’s 2–20 region are critical for SC assembly. In our initial examination of surface-spread meiotic prophase nuclei in *zip1[Δ2–9]* and *zip1[Δ10–14]* strains at the 24 hour time point, we observed many nuclei with extensive SC, as reflected by long linear assemblies of coincident anti-Zip1 and anti-Ecm11-MYC label (Fig 2). In addition, we observed extensive SCs in meiotic nuclei from a strain expressing *zip1[10–14*→*A]*, where each of Zip1’s residues 10–14 is replaced by alanine (Fig 2).

**Fig 3:**
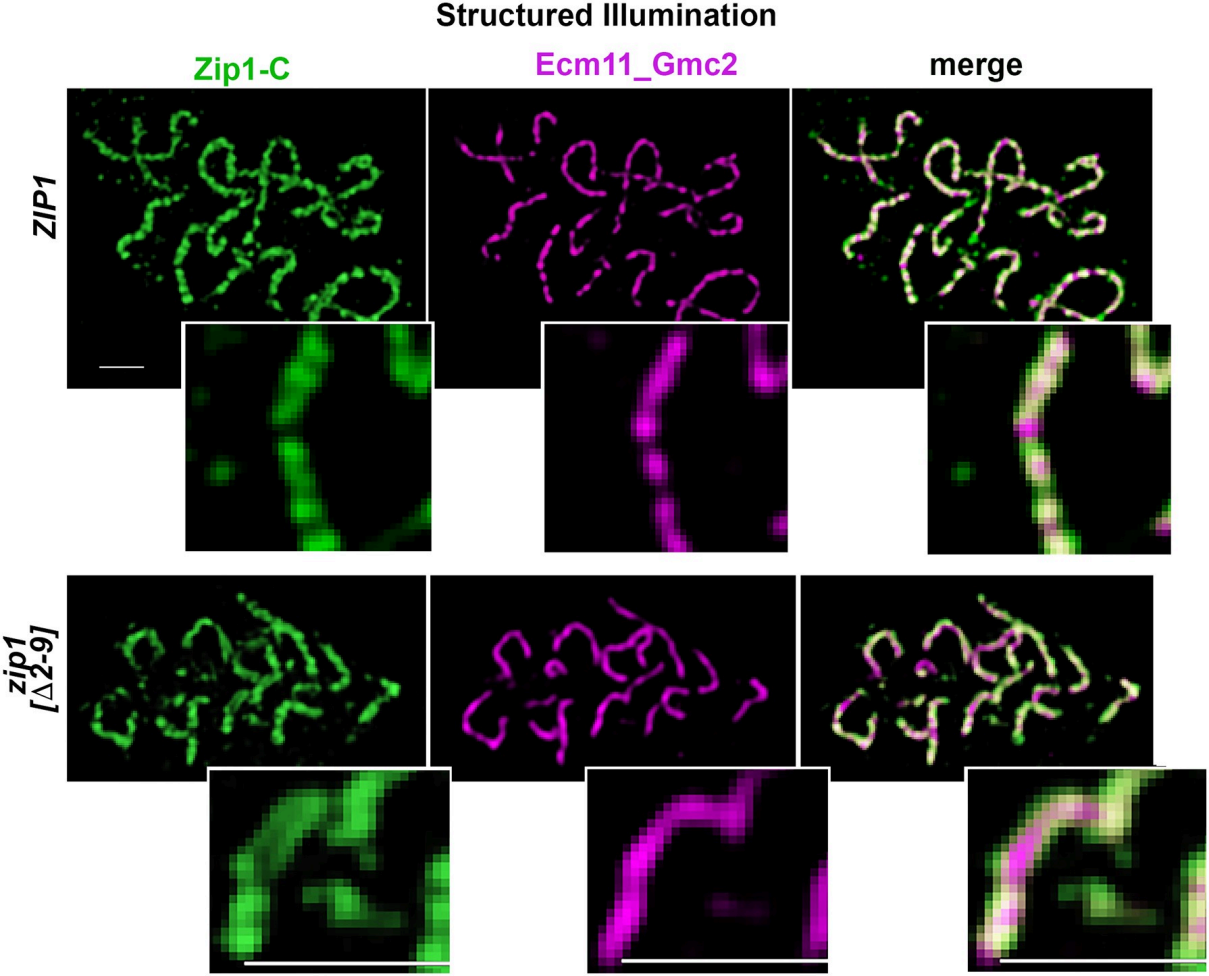
Zip1[Δ2–9] assembles with proper orientation within SCs. Structured illumination was used to probe the organization Zip1 and Ecm11 within linear assemblies in wild type (K1268; top panels) and *zip1[Δ2–9]* (AM4064; bottom panels) strains. Antibodies against the C terminal 264 residues of the Zip1 protein (green) were applied in conjunction with antibodies against a mixture of Ecm11 and Gmc2 partial proteins (magenta; see Methods for antibody information). In surface-spread meiotic nuclei containing wild-type SC (top panels), C terminal Zip1 antibody label often displays a wide ribbon in which parallel tracts are sometimes visible with the resolution power of structured illumination (~120 nm) [13]. Such parallel tracts of Zip1 (see zoomed insets) flank a central narrow band of Ecm11-Gmc2 protein, and reflect Zip1’s orientation within the mature SC, where Zip1’s C termini interface with lengthwise-aligned homologous chromosome axes and Zip1’s N termini orient closer to the SC central element (comprised of Ecm11 and Gmc2). DNA is labeled by DAPI in the experiment but not shown in the figure. Bottom panels show a representative spread where wild-type Zip1 organization is apparent within the SCs built by the Zip1[Δ2–9] protein. Scale bar, 1 μm.

The tripartite SC in budding yeast is assembled by Zip1 transverse filament proteins whose C termini orient toward homologous axes and whose N termini orient nearby to the central element substructure positioned at the midline of the SC. We used structured illumination microscopy (SIM) to ask whether the SC structures in *zip1[Δ2–9]* meiotic nuclei have a canonical, tripartite organization. This tripartite organization can be detected using SIM on surface-spread meiotic chromosomes dually labeled with antibodies that target the C terminal region of Zip1 and SC central element components [13]. With the increased resolution that SIM affords, our anti-Zip1 antibody localizes as a wide ribbon on linear SC structures; one can often observe parallel tracts of Zip1 C termini flanking the central element protein(s) within subsections of such a Zip1 linear element. We observed no detectable difference in the organization of Zip1 and the central element proteins within SCs assembled by wild type Zip1 versus Zip1[Δ2–9] protein: Antibodies targeting the C terminus of Zip1 were observed to flank the SC central element substructure within SCs assembled by Zip1[Δ2–9] (Fig 3).

These observations indicate that, in reciprocal fashion to residues 21–163, residues 2–15 are critically required for Zip1’s MutSγ crossover-promoting activity but dispensable for its capacity to assemble tripartite SC.

In contrast to the robust synapsis observed in *zip1[Δ2–9]* and *zip1[Δ10–14]* strains at the 24 hour time point, extensive coincident linear assemblies of Zip1 and Ecm11 were not detectable in meiotic prophase nuclei from *zip1[Δ15–20]* or *zip1[15–20→A]* mutant strains (Fig 2). Instead, the vast majority of meiotic prophase nuclei from these strains exhibit foci of Zip1 and Ecm11 of varying sizes, which are sometimes, but not always, coincident.

Our phenotypic assessment of three novel non-null *zip1* alleles thus indicates that Zip1’s N terminal twenty residues correspond to adjacent regions that function somewhat independently of one another: residues 2–14 are essential for normal MutSγ-dependent crossovers but dispensable for SC assembly, whereas residues 21–163; [24], and possibly residues 15–20 are less critical for MutSγ crossovers but crucial for SC assembly.

### SC assembles early but may be more labile in MutSγ crossover-deficient but SC-proficient *zip1* and *zip3* mutants

**Fig 4:**
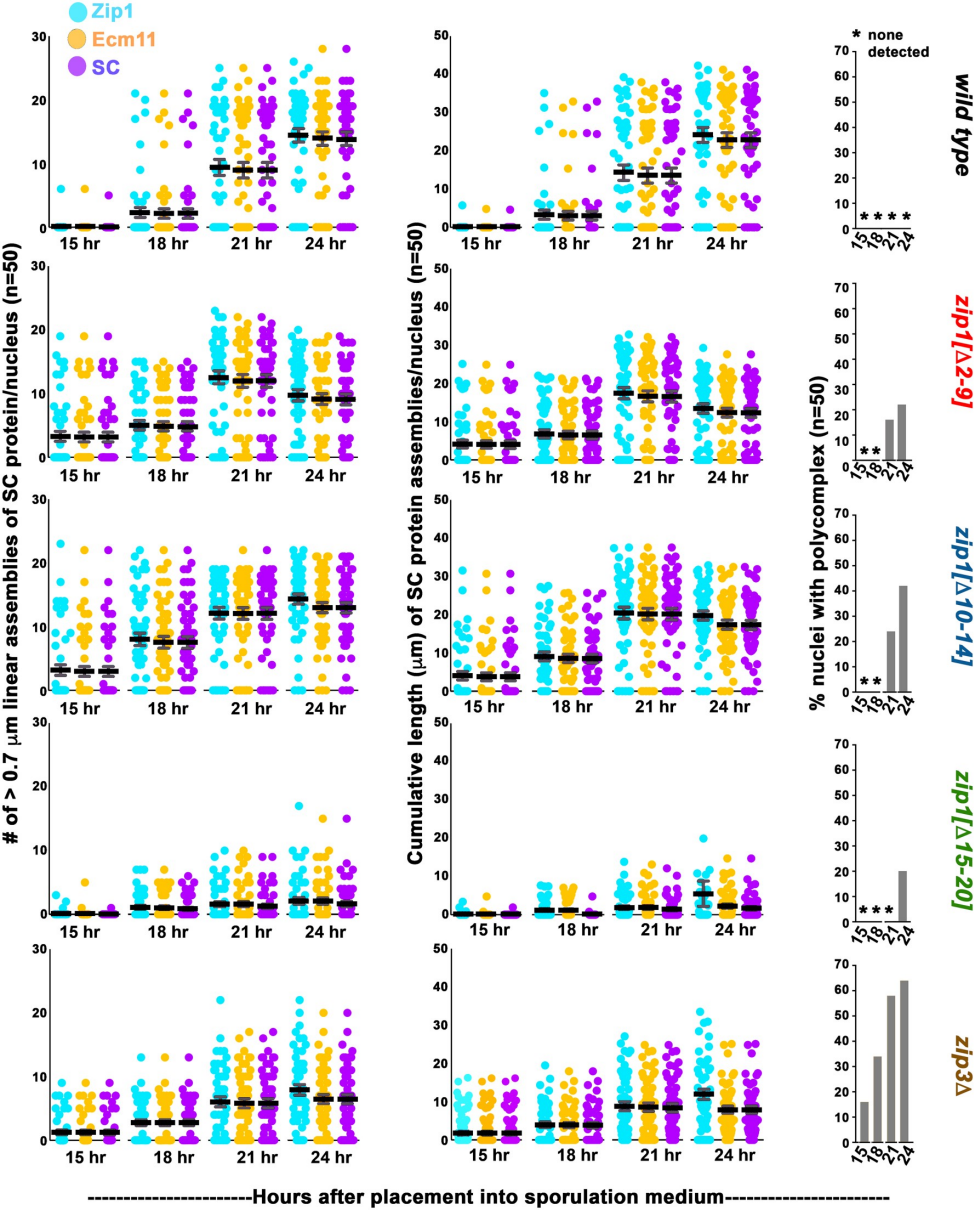
SC assembly requires Zip1 residues 15–20 but not residues 2–14. Each circle in the scatterplots in the far left column represents the number of linear assemblies of Zip1 (blue), Ecm11 (orange) or coincident Ecm11 and Zip1 (purple) detected in surface spread meiotic nuclei of wild-type, *zip1* or *zip3* mutant strains (genotype indicated at the far right; strain names listed in Fig 2 legend), at 15, 18, 21 or 24 hours after placement into sporulation medium (time points indicated on the *x* axis). 50 nuclei were examined for each strain at every individual time point, except for wild-type strains and *zip3* mutant strains at the 15 hour time point, where 125 and 100 nuclei were examined, respectively. Assemblies of SC proteins were considered to be linear if they measured 0.7 μm or greater in length, although some large or adjacent foci potentially were included in these calculations (see arrowhead in Fig 2 and Fig 2 legend). Individual circles in the middle column of scatterplots indicate the cumulative length of the linear assemblies of Zip1 (blue), Ecm11 (orange) or coincident Ecm11 and Zip1 (purple, “SC”) detected in the surface spread nuclei of indicated strains. Dark and light grey bars indicate the mean, and standard error of the mean, respectively. Bar graphs at far right indicate the percentage of nuclei in these datasets that exhibited a Zip1 polycomplex aggregate (see arrow in Fig 2 for an example). The vast majority of polycomplex structures detected contained both Zip1 and Ecm11 proteins. An asterisk indicates zero polycomplex structures detectable in any nuclei at the indicated time point. Raw data for Fig 4 plots is provided in S5 Table.

Our initial 24 hour time point data hinted at a possible deficiency in the number of meiotic nuclei with extensive SC in *zip1[Δ2–9]*, and *zip1[Δ10–14]*, relative to wild-type strains. To explore the possibility that SC assembly occurs with altered timing in these *zip1* mutants, we examined SC abundance in at least 50 meiotic surface-spread nuclei from *zip1[Δ2–9]*, *zip1[Δ10–14]* and *zip1[[Δ15–20]* strains at 15, 18, 21 and 24 hours after placement into sporulation medium. Nuclei were selected solely based on DAPI-stained morphology of the DNA; this method can be used to select nuclei in mid or late meiotic prophase. In addition to a wild-type control strain, our analysis included the *zip3* mutant, which resembles *zip1[Δ 2–9]* and *zip1[Δ10–14]* mutants in its failure to form MutSγ crossovers but proficiency for SC assembly (albeit diminished). We quantified the extent of SC assembly in the selected meiotic nuclei at each time point by measuring the number of linear assemblies of Zip1, Ecm11-MYC, or coincident Zip1-Ecm11-MYC (Fig 4, left column) and the cumulative length of Zip1, Ecm11-MYC, and coincident Zip1-Ecm11-MYC linear structures (Fig 4, center column).

Our analysis revealed that SC cumulative length per meiotic nucleus (as measured by coincident Zip1 and Ecm11-MYC; purple circles in Fig 4, center scatterplots) was only slightly reduced in *zip1[Δ2–9]* and *zip1[Δ10–14]* meiocytes relative to wild type: *zip1[Δ2–9]* and *zip1[Δ10–14]* populations of meiotic nuclei exhibited a maximum of 32 and 38 microns, and on average 17 and 20 microns of cumulative SC length per nucleus, respectively, at the time point displaying the most abundant SC. *ZIP1* control meiotic nuclei exhibited a maximum of 41 and average of 23 microns at the time point with the most abundant SC. *zip3* mutants exhibited a more dramatic reduction in SC cumulative length relative to wild type, with a maximum of 25 and a mean of 8 microns at the time point exhibiting most abundant SC (Fig 4, center column).

Interestingly, SC cumulative length was highest for wild-type populations at the 24 hour time point, while SC cumulative length in *zip1[Δ2–9]*, *zip1[Δ10–14]*, and *zip3* strains peaked at the 21 hour time point in our time course. Moreover, at the earliest time point (15 hour) both the number of SC linear assemblies and SC cumulative length per meiotic nucleus was substantially higher in *zip1[Δ2–9]*, *zip1[Δ10–14]* and *zip3* populations (exhibiting a maximum of 25, 31, 17 microns and mean of 4, 4, 2 microns, respectively, at this time point) relative to the *ZIP1 ZIP3* control population, which exhibited a mean of 0.04 micron of cumulative SC length per nucleus. The difference in SC accumulation at the earliest time point in *zip1[Δ2–9]*, *zip1[Δ10–14]* and *zip3* relative to wild type populations of meiotic nuclei is significant (using an unpaired t-test or the non-parametric Mann Whitney test, the two-tailed *P* value is <0.0001 for each mutant strain) and suggests that SC initiates earlier and/or assembles faster in *zip1[Δ2–9]*, *zip1[Δ10–14]*, and *zip3* meiocytes, relative to wild type. We repeated a similar time course analysis of SC assembly on these strains and observed consistent results (S2 Fig). Early synapsis may have been missed by two earlier analyses of SC assembly in *zip3* because of technicalities: In one study, wild-type nuclei already exhibited substantial SC at the earliest time point examined [45]. An earlier time course spanned appropriate pre-synapsis time points, but only those nuclei with extensive SC were tallied, thus the less extensive SC structures potentially present in the *zip3* mutant at early time points were likely excluded [28].

**Fig 5:**
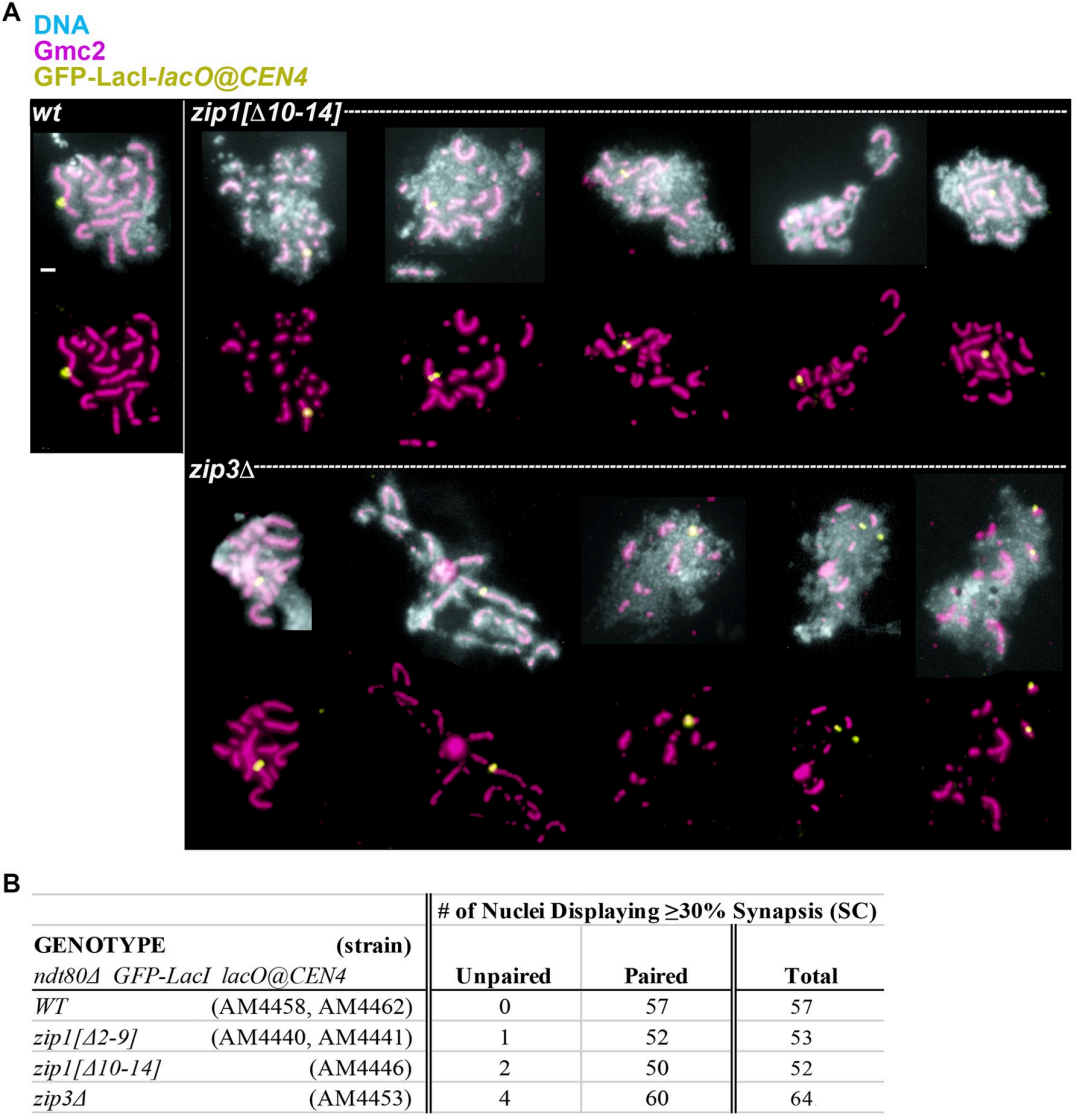
SC in meiotic nuclei from *zip1[Δ2–9]*, *zip1[Δ10–14]*, and *zip3* strains assembles predominantly between homologs. **A)** Representative surface-spread meiotic nuclei from wild-type (AM4458 or AM4462), *zip1[Δ10–14]* (AM4446), or *zip3* (AM4453), strains homozygous for an *ndt80* null allele, a centromere IV-associated *lacO* array, and GFP-LacI. Surface-spread nuclei are labeled with DAPI-labeled DNA (light blue in top images), anti-Gmc2 (magenta), and anti-GFP (green). Meiotic nuclei were examined 23 hours after placement into sporulation medium. Only those nuclei exhibiting approximately 30% minimum SC assembly were assessed for distance between GFP foci. GFP foci were considered paired if they were positioned within 0.5 micron of one another. While most meiotic nuclei examined from all strains exhibit paired GFP signals, occasionally unpaired GFP signals were observed in *zip1[Δ2–9]* (AM4440 or AM4441), *zip1[Δ10–14]* (AM4446), or *zip3* (AM4453) mutants; examples are shown in the two lower right panels (*zip3* strains). Scale bar, 1 μm. **B)** The table lists the number of paired versus unpaired GFP-LacI foci observed in at least fifty meiotic nuclei from *ZIP1 ZIP3* (AM4458 or AM4462), *zip1[Δ2–9]* (AM4440 or AM4441), *zip1[Δ10–14]* (AM4446), or *zip3* (AM4453) strains.

Using a *lacO* array inserted near the centromere of chromosome IV and GFP-LacI expressed in *trans*, we observed that SC assembles between aligned homologous chromosomes in most meiotic nuclei that display moderate to extensive synapsis in *zip1[Δ2–9]*, *zip1[Δ10–14]* and *zip3* strains (n > 50; Fig 5). Thus, while SC assembly initiates early and potentially in a manner that is uncoupled from normal regulatory cues in *zip1[Δ2–9]*, *zip1[Δ10–14]* and *zip3* strains, SC assembly in these mutants remains an event that is triggered downstream of the homologous pairing process.

SC assembly appeared less extensive in *zip1[Δ2–9]*, and *zip1[Δ10–14]* mutants at the 24 hour relative to the 21 hour time point (Fig 4). Although the decrease in cumulative SC at 24 hours is not dramatic, both the range and average cumulative SC length per nucleus shifted lower at the 24 hour time point compared to the 21 hour time point in *zip1[Δ2–9]*, and *zip1[Δ10–14]* strains (Mann Whitney two-tailed *P* = 0.015 for *zip1[Δ2–9]* and 0.071 for *zip1[Δ10–14]*), while cumulative SC increased in wild-type meiotic nuclei at 24 versus 21 hour timepoints (*P* = 0.0014). Furthermore, Zip1 polycomplex aggregates, while undetectable in 100 *zip1[Δ2–9]* and *zip1[Δ10–14]* meiotic nuclei from 15 and 18 hour time points, were observed at the 21 hour time point and were observed even more frequently (30–40% of nuclei, n = 50) at the 24 hour time point (Fig 4, right column). As polycomplex structures tend to assemble when SC assembly is disrupted, the sub-peak level of assembled SC at the 24 and 26 hour time points and the increased occurrence of polycomplex structures together suggest the possibility that SCs assembled in *zip1[Δ2–9]*, and perhaps *zip1[Δ10–14]* strains are less stable than SCs assembled in wild-type strains. The early appearance of polycomplex structures and overall lower extent of assembled SC observed in the *zip3* mutant (ranging from 1.7 microns at 15 hours to 8.4 microns at the time point with peak SC assembly, 21 hours) furthermore suggests that SCs assembled in the absence of Zip3 may be unstable. A replicate time course analysis comparing 21 to 26 hour timepoints gave consistent results (S2 Fig): While the cumulative length of SC in populations of wild-type meiotic nuclei increased between 21 and 26 hour time points (Mann Whitney two-tailed *P* = 0.010), SC decreased between 21 and 26 hours in *zip1[Δ2–9]*, *zip1[Δ10–14]* and *zip3* populations of meiotic nuclei (Mann Whitney two-tailed *P* = 0.0015, <0.0001, and 0.0289 respectively).

Interestingly, a small number of meiotic nuclei with relatively extensive synapsis from *zip1[Δ2–9]*, *zip1[Δ10–14]* and *zip3* mutant strains displayed unpaired GFP-LacI-lacO signals, one or more of which associated with a linear assembly of SC protein (Fig 5). This observation is consistent with the possibility that SC structures prematurely fall apart in these mutants, and either re-assemble on or remain attached to chromosome axes after an original SC breaks down.

**Fig 6:**
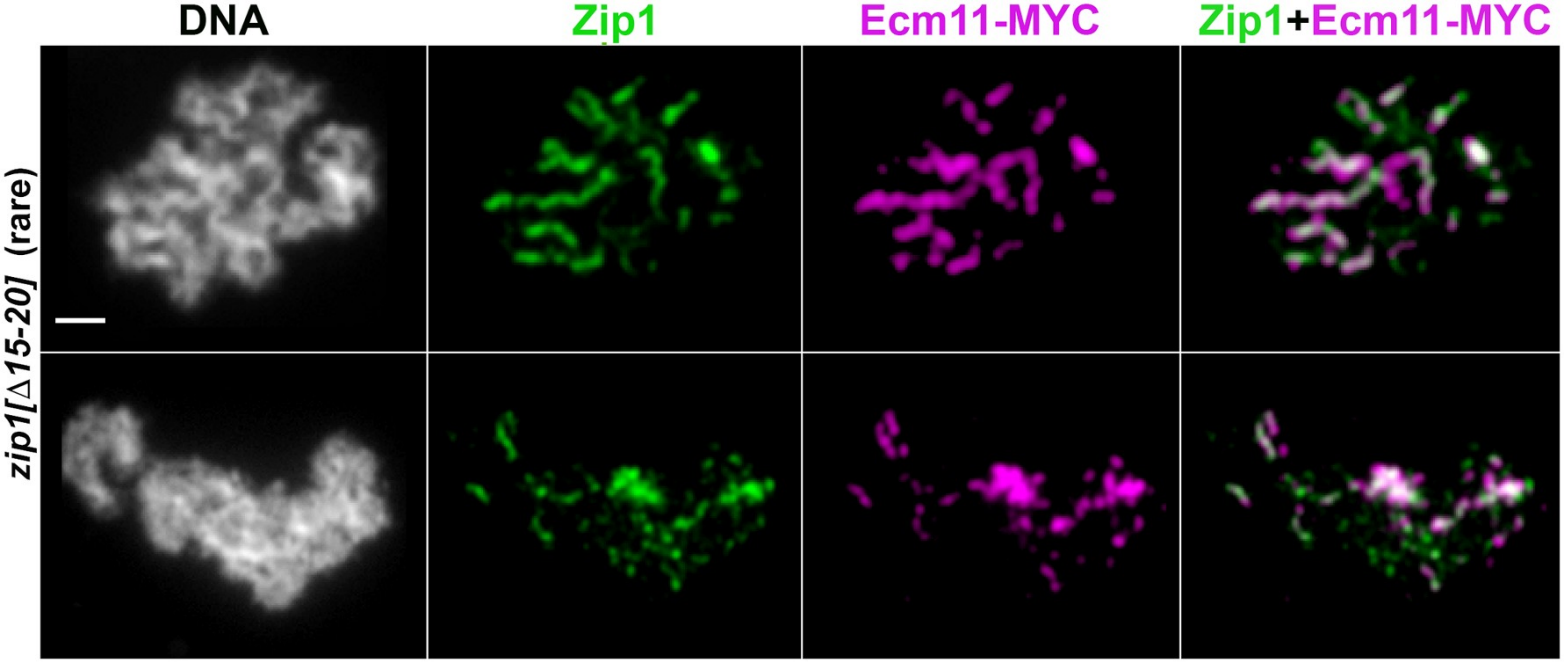
Zip1[Δ15–20] protein occasionally assembles fragile-appearing structures built of coincident Zip1 and Ecm11. While the vast majority of meiotic nuclei from *zip1[Δ15–20]* strains (SYC97) exhibit only abundant and varying-sized foci of Zip1 and Ecm11 (Figs 2 and 4), frail-looking linear assemblies of Zip1 (green) were occasionally observed accompanying similar types of Ecm11 linear assemblies (magenta) on surface-spread meiotic chromosomes (labeled with DAPI, white). The abnormal-looking linear assemblies appear wavy, and often taper at their ends. These assemblies (as well as instances of adjacent large focal deposits of Zip1 and Ecm11) were included in the linear assembly measurements reported in Fig 4. The top row presents the singular rare nucleus, out of the more than 100 nuclei examined, in which these frail linear assemblies were most abundant. We note that unlike the robust linear assemblies of coincident Ecm11 and Zip1 observed in *zip1[Δ2–9]* or *zip1[Δ10–14]* strains, these diffuse linear assemblies do not appear to join lengthwise-aligned chromosomes. Scale bar, 1 μm.

Our time course experiment furthermore confirmed that *zip1[Δ15–20]* meiotic nuclei fail to assemble extensive SC at any point during meiotic prophase (prior to the *ndt80* late meiotic prophase arrest). Out of the 200 *zip1[Δ15–20]* meiotic nuclei analyzed over the time course, zero exhibited robust long linear structures containing coincident Zip1 and Ecm11-MYC. However, large or adjacent foci of coincident Zip1 and Ecm11 were sometimes included in our SC measurements (which recorded any Zip1 or Ecm11 continuous structures with a dimension of 0.7 micron or more), and occasionally a meiotic nucleus displayed linear elements of Ecm11 and Zip1 with a frayed and diffuse appearance (Fig 6). The average cumulative length of SC per nucleus detected in *zip1[Δ15–20]* populations was 1.7 microns at the peak time point (24 hours; Fig 4). Zip1 polycomplex structures were observed only at the 24 hour time point in *zip1[Δ15–20]* strains (Fig 4, right column).

### Crossover-deficient, synapsis-proficient *zip1* mutants initiate SC assembly from both centromeric and interstitial chromosomal sites

**Fig 7:**
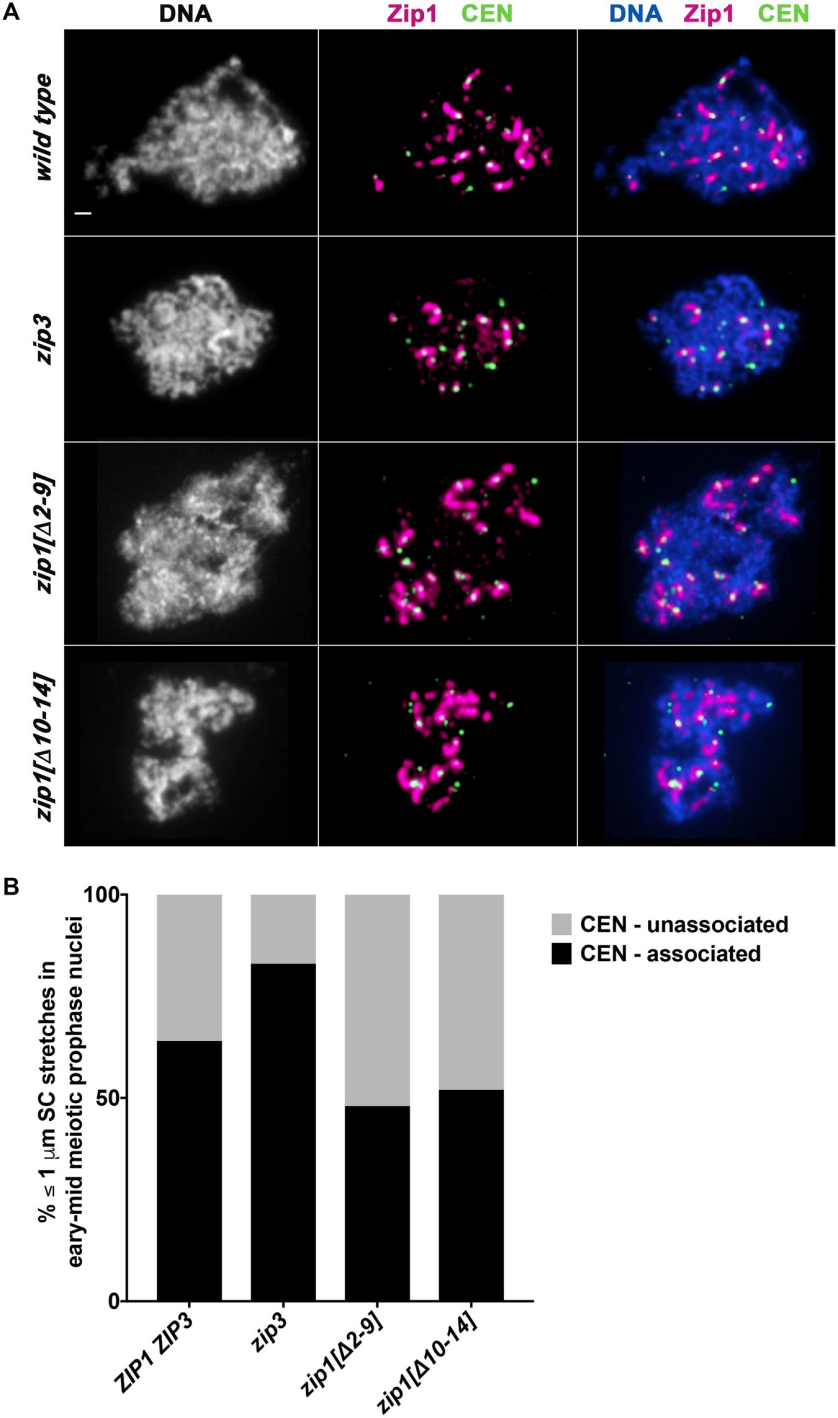
SC comprised of Zip1[Δ2–9] or Zip1[Δ10–14] protein initiates at both centromeric and non-centromeric chromosomal sites. **A)** Representative surface-spread meiotic nuclei from wild-type (AM4203; top row), *zip3* (AM4204; second row), *zip1[Δ2–9]* (AM4231; third row) or *zip1[Δ10–14]* (AM4175; bottom row) strains producing the centromere-associated Ctf19-MYC protein. Surface-spread nuclei are labeled with DAPI-labeled DNA (white), anti-Zip1 (magenta), and anti-MYC (green). While all strains at this early stage of synapsis exhibit a large number of short Zip1 assemblies associated with a centromere, fewer examples of ≤1 μm Zip1 assemblies without an associated centromere were observed in *zip3* strains, relative to wild-type, *zip1[Δ2–9]* or *zip1[Δ10–14]* strains. Scale bar, 1 μm. Quantitation of the number of ≤1 μm Zip1 assemblies with or without an associated Ctf19-MYC signal is shown in the bar graphs in **(B)**, where light shading represents the percentage of total ≤1 μm Zip1 assemblies in each strain that are unassociated with a centromere signal. In wild-type strains, 23 out of 36 ≤1 μm Zip1 assemblies (in 10 surface-spread nuclei) were associated with a centromere; In *zip3* strains, 29 out of 35 ≤1 μm Zip1 assemblies (in 11 nuclei) were associated with a centromere; In *zip1[Δ2–9]* strains, 35 out of 73 ≤1 μm Zip1 assemblies (in 21 nuclei) were associated with a centromere; In *zip1[Δ10–14]* strains, 27 out of 52 ≤1 μm Zip1 assemblies (in 11 nuclei) were associated with a centromere. A Fishers Exact Test found no significant difference between the proportion of centromere-associated Zip1 stretches in wild-type versus *zip3* in this data set (two-tailed *P* value = 0.107), but did find a significant difference between *zip1[Δ2–9]*, and *zip3* (two-tailed *P* value = 0.0007), and between *zip1[Δ10–14]* and *zip3* (two-tailed *P* value = 0.0033).

While the earliest SC assembly events that occur during meiosis in budding yeast have been found to preferentially initiate at centromeres [29], SC assembly events are also associated with non-centromeric sites (presumably recombination sites) in wild-type meiotic nuclei at intermediate stages of synapsis. In the *zip3* mutant, by contrast, new SC assembly events associate predominantly with centromeres at both early and later meiotic prophase stages [29]. As the SC assembly and meiotic crossover phenotypes in *zip1[Δ2–9]* and *zip1[Δ10–14]* strains resemble the phenotypes found in *zip3* mutant meiotic cells, we asked whether nascent SC assembly events in *zip1[Δ2–9]* and *zip1[Δ10–14]* meiotic nuclei associate with centromeres more often than wild-type nuclei at intermediate stages of synapsis. Surface-spread meiotic chromosomes from 15, and 18 hour time points were co-labeled with antibodies that target Zip1, and that target the MYC epitope that is fused to the Ctf19 centromere protein in these strains. In order to enrich for new, singular SC assembly events in our analysis, we identified the total number of Zip1 linear stretches measuring between 0.7–1.0 micron in length, and measured the number that are directly adjacent to (overlapping) a Ctf19-MYC focus. We found that 83% (29 out of 35) of such short SC structures were associated with a Ctf19-MYC focus in *zip3* meiotic nuclei, consistent with previously-published data [29]. By contrast, 48%, 52% and 64% (35/73, 27/52, and 23/36) of short Zip1 linear structures were associated with Ctf19-MYC in *zip1[Δ2–9]*, *zip1[Δ10–14]*, or *ZIP1 ZIP3* meiocytes, respectively (Fig 7). Thus, in contrast to *zip3* mutants, *zip1[Δ2–9]*, and *zip1[Δ10–14]* mutants display a wild-type capacity to initiate SC assembly from non-centromeric sites on meiotic chromosomes.

### Adjacent regions within Zip1’s first twenty residues have opposing effects on the SUMOylation of an SC central element protein

Humphryes et. al (2013) demonstrated that SUMOylated Ecm11 is required for SC assembly, and that the central element component Gmc2, transverse filament Zip1, along with SIC proteins Zip2, Spo16 and Zip4 (but not Zip3) are required for robust Ecm11 SUMOylation during meiosis. This report also revealed that Ecm11 is hyper-SUMOylated in mutants missing the putative SUMO E3 ligase and SIC protein, Zip3. To ask whether the N terminal twenty residues of Zip1 are required for its capacity to regulate Ecm11 SUMOylation, we evaluated the abundance of Ecm11 forms in meiotic extracts from strains homozygous for *ZIP1*, *zip1[Δ2–9]*, *zip1[Δ10–14]*, *zip1[Δ15–20]*, a *zip1* null, or a *zip3* null allele. These strains also are homozygous for the *ndt80* mutation, in order to allow our asynchronous meiotic cultures to accumulate (by 24 hours after placement into sporulation medium) at a mid-late meiotic prophase stage, when the Ecm11 SUMOylation that accompanies synapsis is at a maximum [42].

**Fig 8:**
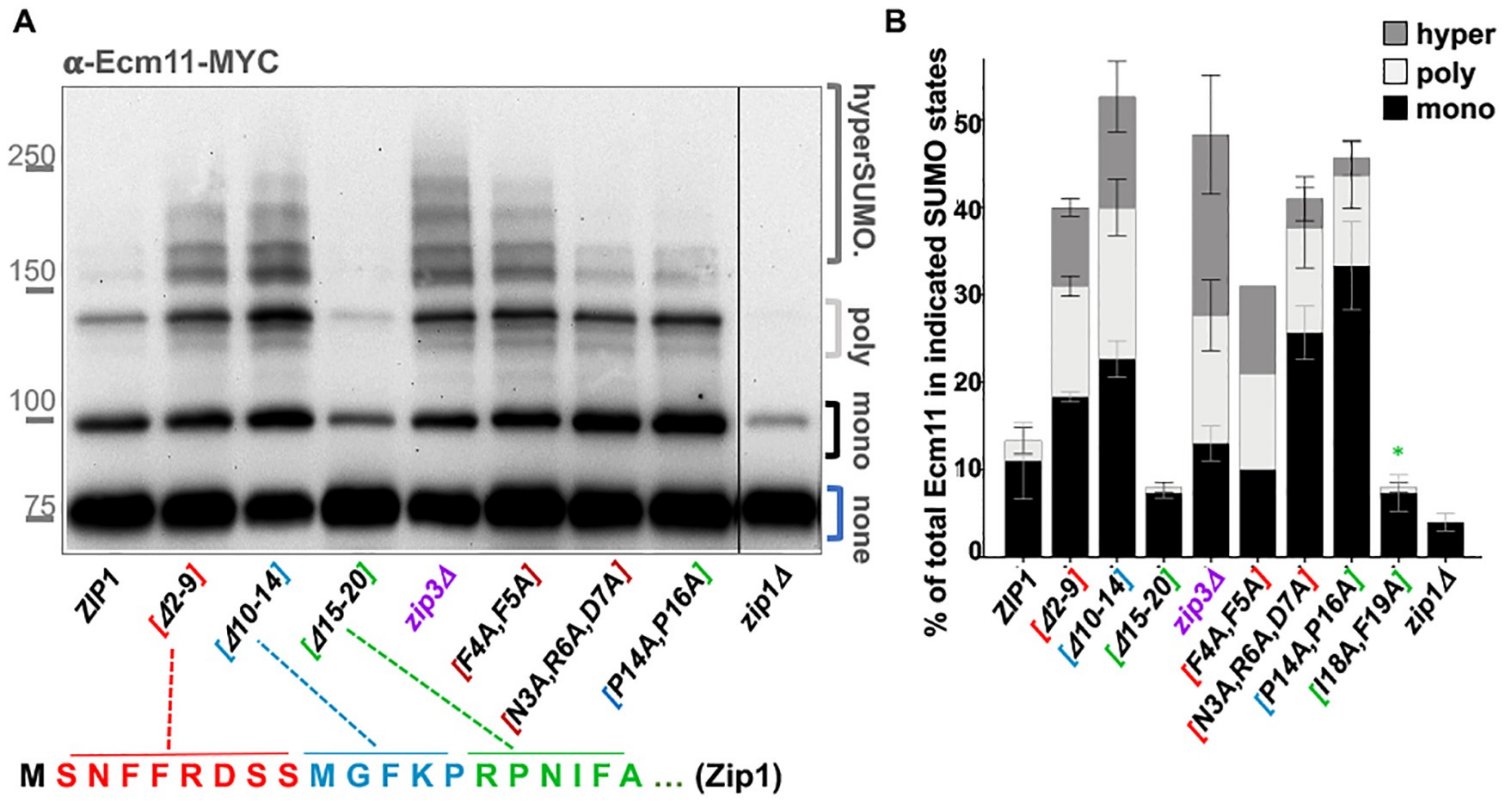
Zip1 residues 15–20 promote, while residues 2–14 limit, SUMOylation of the SC central element protein Ecm11. **A)** A representative Western blot using an anti-MYC antibody reveals unSUMOylated, mono-SUMOylated, poly-SUMOylated and hyper-SUMOylated forms of Ecm11-MYC in meiotic extracts prepared from *ZIP1 ZIP3*, *zip3*, or various *zip1* mutant strains (protein alterations caused by each *zip1* allele is indicated on the *x* axis). All strains carry an *ndt80* null allele, which causes a meiotic arrest that ensures maximal enrichment of mid-meiotic prophase stage cells at 24 hours after placement into sporulation medium [44]. Meiotic extracts were prepared at 24 hours after placement into sporulation media, as previously described [13, 38]. Strains included in this analysis are: AM2712 (*ZIP1*), MP39 (*zip1[Δ2–9])*, SYC96 (*zip1[Δ10–14])*, SYC97 (*zip1[Δ15–20])*, AM3719 (*zip3)*, AM3662 (*zip1[F4A*,*F5A])*, AM3628 (*zip1[N3A*,*R6A*,*D7A])*, AM3656 (*zip1[P14A*,*P16A])*, AM2784 (*zip1Δ)*, K986 (*zip1[I18A*,*F19A])*. **B)** The stacked bar graph plots the percentage of mono-SUMOylated (dark shaded bar), poly-SUMOylated (light bar), or hyper-SUMOylated (gray shaded bar) forms of Ecm11-MYC detected in each strain at 24 hours after placement into sporulation media. The absence of a light or gray bar in some strains indicates that this form of Ecm11 was detected in less than 1% of the total population. Error bars represent the range of values from three independent meiotic cultures, except in the case of the *zip1[F4A*, *F5A]* strain, where only one experiment was performed. Data plotted is listed in S6 Table. * Note *zip1[I18A*, *F19A]* is not present in the blot shown in (A).

A Western blot can readily detect three forms of Ecm11-MYC in protein extracts from meiotic cells homozygous for MYC-tagged Ecm11 [13, 35, 38]. UnSUMOylated Ecm11-MYC migrates near the 75 kD marker on a protein gel, whereas monoSUMOylated and polySUMOylated Ecm11-MYC is positioned near the 100 kD and 150 kD positions, respectively. HyperSUMOylated Ecm11-MYC, which is abundant in *zip3* meiotic extracts, migrates at various positions between the 150 kD and 250 kD markers (Fig 8A).

We found the proportion of SUMOylated Ecm11-MYC in *ZIP1 ZIP3 ndt80* meiotic extracts at the 24 hour time point to be, on average, 14%, wherein 11% of total Ecm11-MYC was of the monoSUMOylated form and 3% was of the polySUMOylated form (three replicates; Fig 8B). Consistent with prior results [13, 38], *zip1* null strains exhibited a relatively low level of SUMOylated Ecm11: An average of 4% of total Ecm11-MYC was of the monoSUMOylated form, while polySUMOylated Ecm11-MYC was below levels of detection (less than 1%; Fig 8B). Again consistent with prior findings [38], *zip3* meiotic extracts exhibited not only an elevated level of polySUMOylated Ecm11-MYC (15% of total Ecm11-MYC, on average, over three replicates), but also an abundance of hyperSUMOylated Ecm11 (19% of total Ecm11-MYC, on average, over three replicates; Fig 8B).

We found that residues 15–20 are important for Zip1’s capacity to promote Ecm11 SUMOylation. In *zip1[Δ15–20]* meiotic extracts at the 24 hour time point, on average only 8% of total Ecm11 was of a SUMOylated form (Fig 8B). Given the SC assembly defect of *zip1[Δ15–20]* strains, this result bolsters a direct correlation between Ecm11 SUMOylation and SC assembly.

In striking contrast, cells expressing the *zip1[Δ2–9]* or *zip1[Δ10–14]* alleles exhibit robust levels of Ecm11 SUMOylation as well as the hyperSUMOylated forms of Ecm11 that are characteristic of the *zip3* mutant. An average of 40% of Ecm11-MYC was SUMOylated in *zip1[Δ2–9]* meiotic extracts at the 24 hour time point, with 9% in the hyperSUMOlated form (Fig 8B). Likewise, an average of 54% of Ecm11-MYC was SUMOylated in *zip1[Δ10–14]* meiotic extracts, with 13% in the hyperSUMOylated form (Fig 8B).

It has been proposed that the hyperSUMOylated forms of Ecm11 that occur in *zip3* mutant meiotic cells correspond to Ecm11 linked to poly-SUMO branched chain structures of various sizes and shapes [38, 46]. The accumulation of such extensively SUMOylated Ecm11 protein in *zip1[Δ2–9]* and *zip1[Δ10–14]* mutants indicates that residues within the 2–14 region of Zip1 are dispensable for Ecm11 SUMOylation *per se*, but regulate the extent and/or manner of Ecm11 SUMOylation. Zip1’s residues 2–14 appear to control similar aspects of Ecm11 SUMOylation as the putative SUMO E3 ligase, Zip3, raising the possibility that Zip1’s N terminal residues regulate Ecm11 SUMOylation in part through an interaction with Zip3.

### The phenotype of two- or three- residue alterations within Zip1’s N terminus resemble corresponding small internal deletion alleles

We found that *zip1* alleles encoding proteins with alanine substitutions in place of dual or triple residues within Zip1’s N terminus exhibit the distinguishing Ecm11 SUMOylation and synapsis phenotypes of corresponding internal deletion *zip1* alleles. *zip1[N3A*, *R6A*, *D7A]*, *zip1[F4A*, *F5A]*, and *zip1[P14A*, *P16A]* exhibited elevated levels of SUMOylated Ecm11 during meiosis, reminiscent of the *zip1[Δ2–9]* and *zip1[Δ10–14]* mutants, although we note that *zip1[N3A*, *R6A*, *D7A]* and *zip1[P14A*, *P16A]* strains exhibit a particular abundance of monoSUMOylated relative to polySUMOylated and hyperSUMOylated Ecm11, which differs slightly from the distribution of SUMOylated Ecm11 forms in *zip1[F4A*, *F5A]*, *zip1[Δ2–9]*, *zip1[Δ 10–14]* or *zip3* mutants (Fig 8B). By contrast, meiotic extracts from *zip1[I18A*, *F19A]* strains exhibit a dramatic reduction in SUMOylated Ecm11, reminiscent of meiotic extracts from *zip1[Δ15–20]* and *zip1* null strains (Fig 8B).

**Fig 9:**
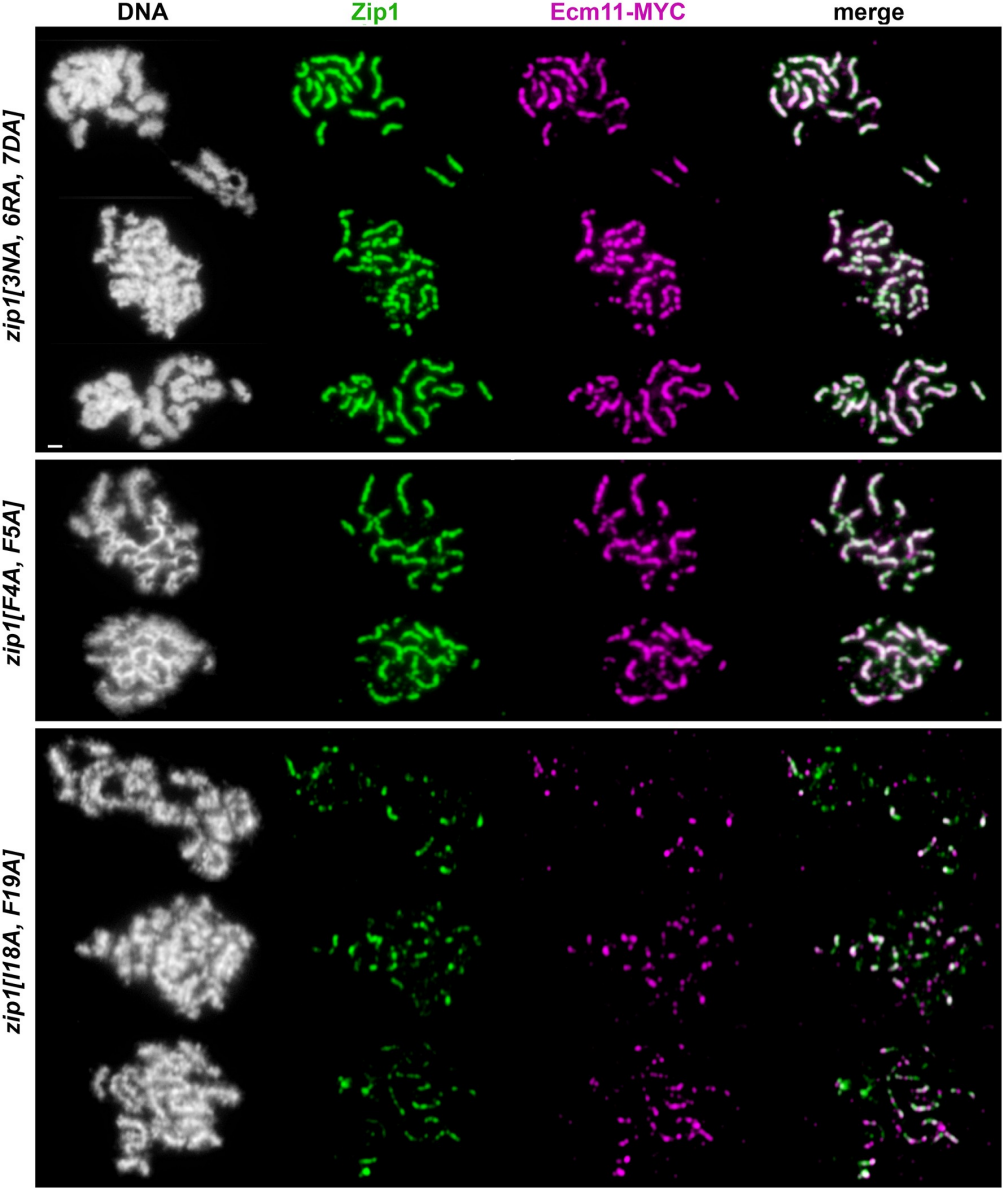
*zip1[N3A*, *R6A*, *D7A]* and *zip1[F4A*, *F5A]* and *zip1[I18A*, *F19A]* mutants resemble corresponding small deletion alleles with respect to synapsis. Panels show representative surface-spread mid-meiotic prophase nuclei from *zip1[N3A*, *R6A*, *D7A]* (K969; top panel including 3 rows), *zip1[F4A*, *F5A]* (AM4067; middle panel including two rows) and *zip1[I18A*, *F19A]* (K985; bottom panel including three rows) mutants, with genotypes indicated at left. All strains carry an *ndt80* null allele, which allows meiotic cultures to accumulate at mid-late prophase stages when full-length SCs are normally present. Mid-meiotic prophase chromosomes are stained with DAPI to label DNA (white), anti-Zip1 (green), and anti-MYC to label the epitope-tagged Ecm11 expressed in these strains (magenta). The merge between Zip1 and Ecm11-MYC channels is shown in the final column.

We also found that *zip1[N3A*, *R6A*, *D7A]* and *zip1[I18A*, *F19A]* exhibit SC assembly phenotypes that are generally reminiscent of corresponding internal deletion *zip1* alleles. Linear stretches of coincident Zip1 and Ecm11-MYC were often detectable on surface-spread meiotic chromosomes from *zip1[N3A*, *R6A*, *D7A] ndt80* and *zip1[F4A*, *F5A] ndt80* strains at multiple time points in a meiotic time course, while such extensive SC structures were absent from meiotic chromosomes in *zip1[I18A*, *F19A] ndt80* strains at all time points (Fig 9).

We furthermore found that meiotic crossovers are reduced in *zip1[N3A*, *R6A*, *D7A]*, *zip1[F4A*, *F5A]*, and *zip1[I18A*, *F19A]* point mutants in a manner that resembles the corresponding deletion strain (S2 Table). Specifically, *zip1[F4A*, *F5A]* exhibited the most dramatic deficit in meiotic crossovers, and crossovers in this mutant are not further reduced by removal of *MSH4*, indicating that *zip1[F4A*, *F5A]* meiotic cells lack MutSγ-mediated crossovers (S2 Table). Crossovers did further diminish from their intermediate level when *MSH4* was removed from *zip1[I18A*, *F19A]* mutants (S2 Table).

The phenotypes of these novel *zip1* dual- and triple-residue substitution alleles strengthen the idea that Zip1’s first twenty residues encompass both crossover recombination and SC assembly functionalities and that adjacent sites within this region maintain different and independent roles in regulating synapsis.

### Residues within the 2–14 region influence Zip1’s capacity to interface with Zip3 at polycomplex structures

The shared phenotypes of *zip1[Δ2–9]*, *zip1[Δ10–14]* and *zip3* mutants prompted us to wonder whether the N terminus of Zip1 directly or indirectly interacts with the Zip3 protein. Prior evidence for an interaction between Zip1 and Zip3 includes the observation that Zip3 is detected throughout Zip1 polycomplex structures that assemble in contexts where SC assembly fails [28, 34]. To explore the possibility that Zip1’s N terminus mediates an interaction with the Zip3 protein, we examined the distribution of Zip3 at Zip1 polycomplex structures assembled in *spo11* meiotic cells, which fail to initiate recombination and thus also SC assembly [45, 47].

**Fig 10:**
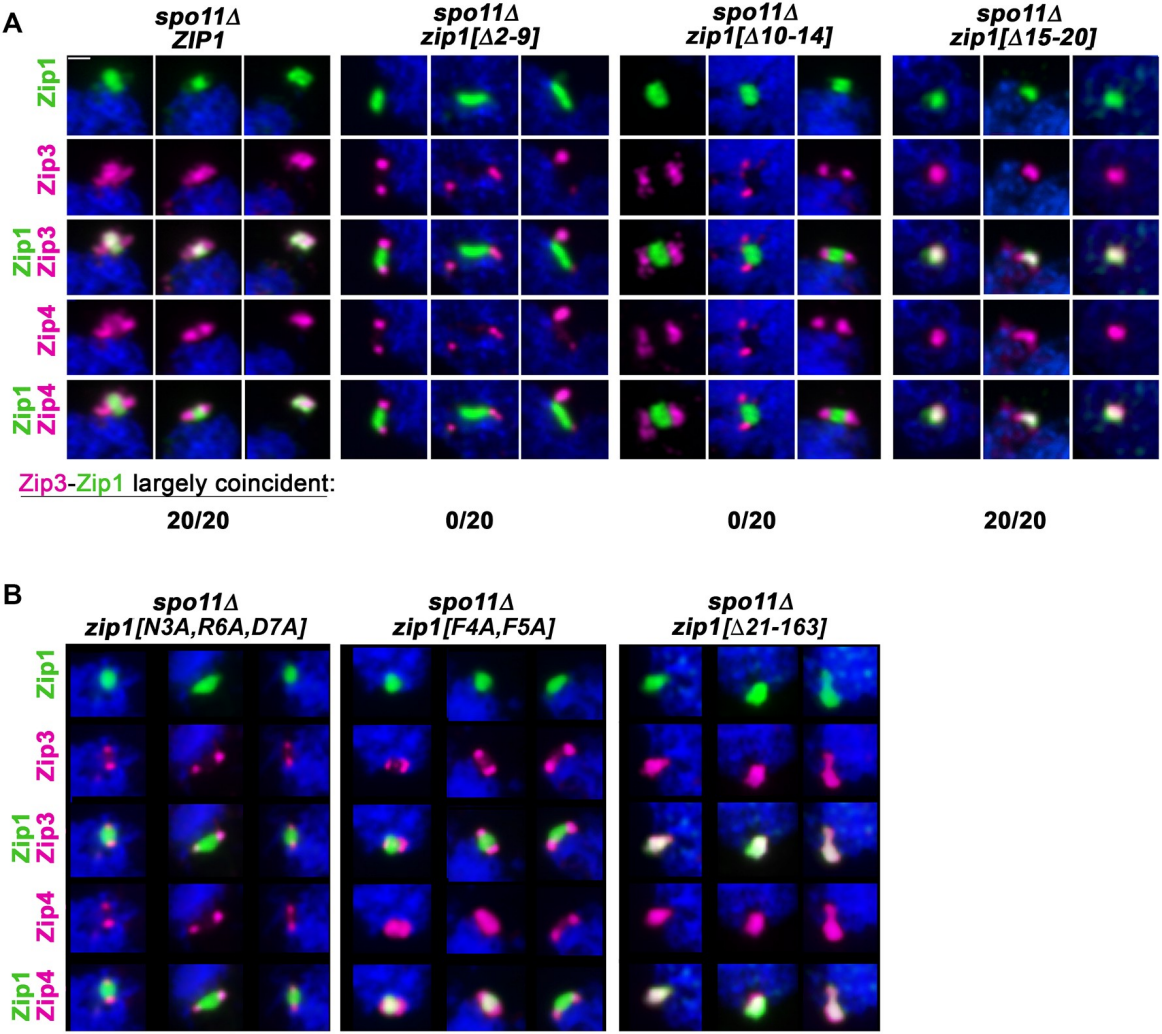
Zip1 residues 2–14 promote the localization of Zip3 to Zip1 polycomplex structures. **A)** Four groups of panels each show three representative images of Zip1 polycomplex structures in *spo11* meiotic prophase nuclei expressing either wild-type *ZIP1* (AM4174; far left group), *zip1[Δ2–9]* (AM4253; second group), *zip1[Δ10–14]* (AM4173; third group), or *zip1[Δ15–20]* (AM4256; far right group). Zip1 polycomplex is shown in green (first row) on surface-spread meiotic prophase nuclei. The localization of Zip3-MYC (second row, magenta) and Zip4-HA (fourth row, magenta) is also assessed. Merged images between either Zip3-MYC and Zip1 or Zip4-HA and Zip1 are shown in the third and fifth rows, respectively. The number of polycomplexes (n = 20) for which Zip3 is nearly fully coincident with Zip1 is displayed at the bottom of the corresponding strain’s images (n = 20). (**B**) Three groups of panels each show three different surface spread *spo11* meiotic nuclei with a Zip1 polycomplex structure (labeled by anti-Zip1; green, top row). The localization of Zip3-MYC (second row, magenta) and Zip4-HA (fourth row, magenta) is also assessed on these surface-spread meiotic prophase nuclei. Merged images between either Zip3-MYC and Zip1 or Zip4-HA and Zip1 are shown in the third and fifth rows, respectively. Nuclei at the far left correspond to strains homozygous for *zip1[N3A*, *R6A*, *D7A]* (AM4350); the middle panel corresponds to strains homozygous for *zip1[F4A*, *F5A]* AM4340; and nuclei in the far right panel correspond to strains homozygous for *zip1[Δ21–163]* (AM4343). Scale bar, 1 μm.

Of the polycomplex structures assembled by wild-type Zip1 and Zip1[Δ15–20] protein, 100% (20/20) exhibited Zip3-MYC distributed uniformly across the entire structure (Fig 10A). Frequently, additional “capping” structures of coincident Zip3-MYC and Zip4-HA protein flank the Zip1 polycomplex, as has been reported previously [34]. Intriguingly, however, among more than 20 meiotic nuclei examined from *spo11 zip1[Δ2–9]* and *spo11 zip1[Δ10–14]* strains, Zip3-MYC was completely absent from the bulk of the Zip1 polycomplex structure (Fig 10A). Instead, Zip3-MYC co-localized with Zip4-HA in the capping configuration at opposite ends of the polycomplex. Often these “capping” structures of coincident Zip3-MYC and Zip4-HA were observed at a substantial distance away from the polycomplex aggregate of Zip1 protein.

We furthermore found that Zip3 is diminished at polycomplexes assembled by Zip1[N3A, R6A, D7A] or Zip1[F4A, F5A] protein (Fig 10B), although the absence of Zip3 from the bulk of these Zip1 polycomplex structures is less dramatic than what is observed at Zip1[Δ2–9] or Zip1[Δ10–14] polycomplex. Finally, as expected based on the robust localization of Zip3 to Zip1[Δ15–20] polycomplex, we found that Zip3 also localizes uniformly throughout polycomplexes built of Zip1[Δ21–163] protein (Fig 10B).

These data indicate that, at least in the context of polycomplex structure, Zip1’s residues 2–14 mediate a direct or indirect interaction with the pro-crossover and putative E3 SUMO ligase protein, Zip3.

### Residues within Zip1’s 2–14 region are critical for Zip3 recruitment to recombination initiation sites

Zip3 and other SIC proteins (such as Zip2, Zip4 and Spo16) form foci that co-localize with MutSγ along aligned homologous chromosomes at mid meiotic prophase [28, 34]. Consistent with the notion that such Zip3 foci mark recombination intermediates, Zip3 has been detected at DSB hotspots using chromatin immunoprecipitation (ChIP) [37, 48]. Zip1 was found to be required for the recruitment of Zip3 to the DSB sites examined, thus we asked whether the capacity of Zip1 to recruit Zip3 to DSB sites relies on the N terminal residues of Zip1 that facilitate Zip3’s localization to Zip1 polycomplex.

We performed ChIP in conjunction with quantitative PCR (ChIP-qPCR) on meiotic cell extracts from *ZIP1*, *zip1[Δ2–9]*, *zip1[Δ10–14]*, and *zip1* null strains expressing a Zip3 protein with three copies of the FLAG epitope fused to its C terminus. Strains for this experiment were built in the SK1 genetic background, to ensure maximal synchrony over a meiotic time course (SK1 strains enter and/or progress through meiosis more synchronously than the BR strain background used for all other experiments in this study). ChIP-qPCR was performed at multiple time points during sporulation, and the time course experiment was performed in duplicate for each strain except for the *zip1* null negative control, where the single experiment performed gave results that are consistent with prior published data [48].

We examined Zip3-6xHIS-3xFLAG association with chromosomal sites corresponding to three known DSB hotspots, a centromere, or the chromosome axis [37, 48]. Sequences enriched for Rec8 that are embedded in the proteinaceous chromosome axis are generally anti-correlated with DSB sites, but are thought to associate with DSB repair intermediates, according to a “loop-tether” model for DSB formation in budding yeast [49]. A sequence internal to the large *NFT1* open reading frame was previously found to be devoid of Zip3 binding [50, 51] and thus served as a negative control for Zip3 enrichment.

**Fig 11:**
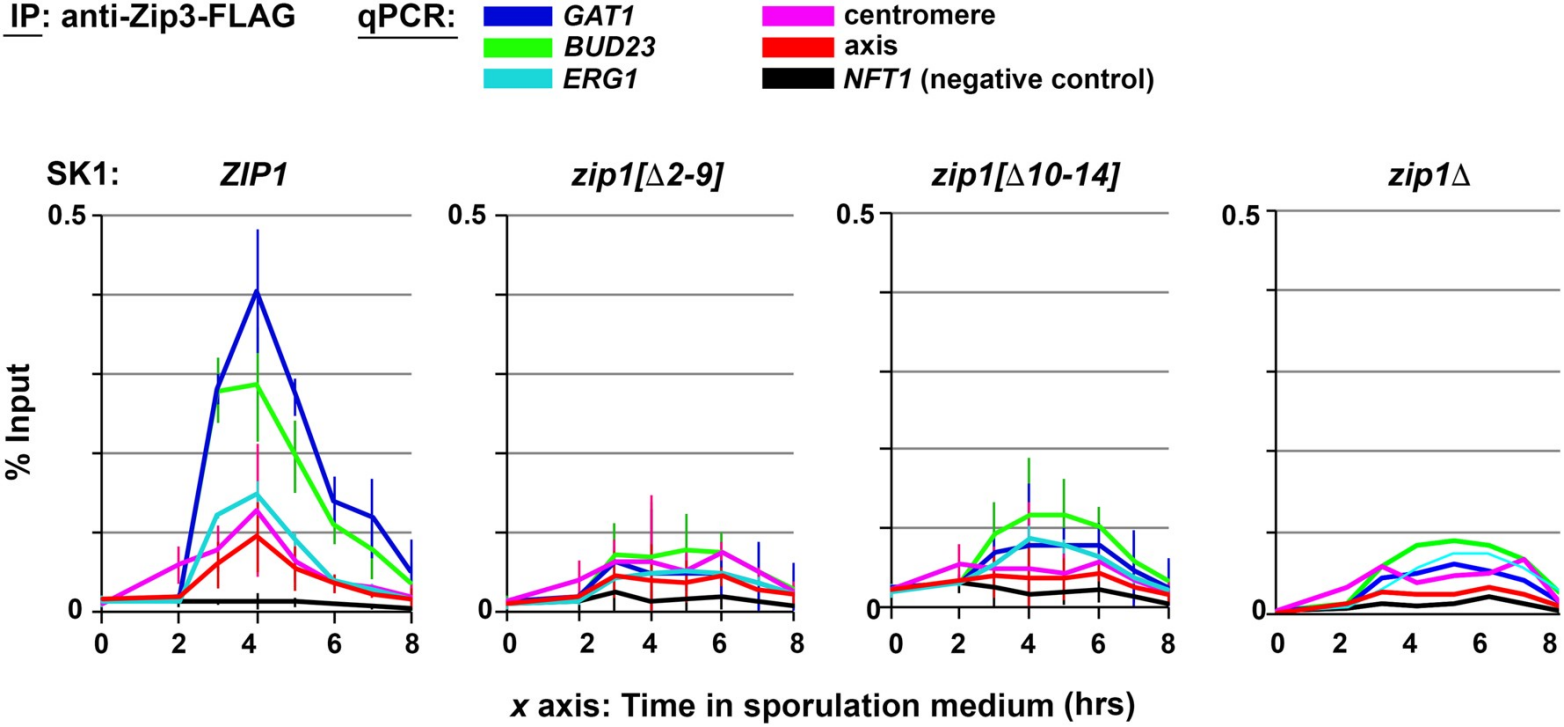
Residues 2–14 are required for Zip1’s capacity to recruit Zip3 to recombination initiation sites. Chromatin immunoprecipitation (ChIP) followed by quantitative PCR (qPCR) to monitor the association of Zip3-FLAG with three DSB sites (*GAT1*, *BUD23*, *ERG1*, dark blue, green and light blue lines, respectively) as well as to centromere and axis sites (magenta and red lines, respectively) in SK1 strains carrying *ZIP1* (ORD9670), *zip1[Δ2–9]* (AM3946/VBD1872), *zip1[Δ10–14]* (AM3951/VBD1873), or *zip1*Δ (ORD9689) alleles. *x* axes indicate number of hours after placement in sporulation medium. The relative abundance of indicated chromosomal sites detected by qPCR in Zip3-FLAG immunoprecipitates is expressed as a percentage of the abundance of each site detected in the input, prior to immunoprecipitation. Values are the average ± standard deviation from two independent experiments. A single experiment was performed in the case of the *zip1* null strain. Data plotted is listed in S6 Table.

In strains carrying wild-type *ZIP1* within two hours after placement of into sporulation medium, centromeric DNA was more abundant in Zip3 immunoprecipitates relative to axis or DSB site DNA, consistent with previously published results [37, 48]. Between two and four hours after placement into sporulation medium, DNA sequences corresponding to chromosome axis sites and three DSB hotspots (*GAT1*, *BUD23*, and *ERG1*; Fig 11) significantly increased their abundance within Zip3 immunoprecipitates, reflecting Zip3 recruitment to these chromosomal sites. Zip3 localization to all sites peaked at the 4 hour time point in *ZIP1* meiotic cells, which corresponds to maximal DSB activity at the *BUD23* locus in this SK1 strain background [48]. At this four hour time point, Zip3 enrichment was found to be two to three fold greater at *GAT1* and *BUD23* compared to DNA sequences at the chromosome axis (Fig 11). Consistent with DSB repair timing in this genetic background, Zip3 enrichment at all sites dramatically diminished between four and six hours, and was at pre-meiotic levels by eight hours after placement in sporulation medium. Consistent with prior findings, Zip3 was virtually undetectable at DSB, axis and centromere sites in the *zip1* null strain (in which the *ZIP1* ORF is deleted; Fig 11; [48]).

Similar to a *zip1* null strain, little Zip3 was detectable at any of the three DSB sites examined, nor at the axis or centromere site, in *zip1[Δ2–9]* or *zip1[Δ10–14]* strains (Fig 11). The phenotype of *zip1[Δ2–9]* in this experiment appeared indistinguishable from the *zip1* null, whereas slight Zip3 enrichment was detected at the *BUD23* DSB site in the *zip1[Δ10–14]* meiotic time course. These data indicate that the capacity of Zip1 to recruit Zip3 to DSB sites during meiosis relies on Zip1’s N terminal residues 2–14.

**Fig 12:**
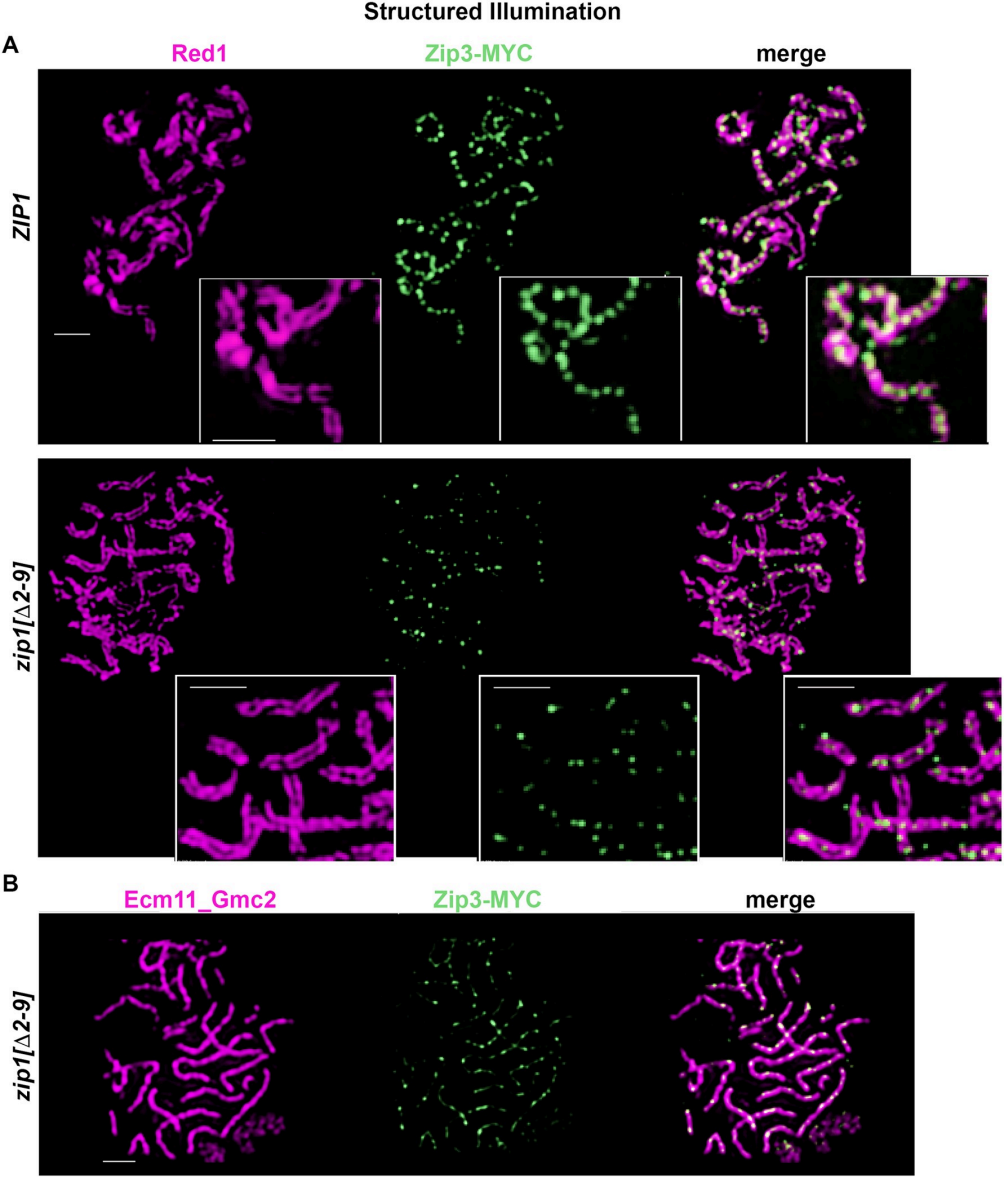
Zip3-MYC foci localize to the SC central element sub-structure in wild-type meiotic nuclei and are diminished in number and intensity in *zip1[Δ2–9]* and *zip1[Δ10–14]* strains. Images show representative surface-spread meiotic nuclei labeled with antibodies against the chromosomal axis protein Red1 (magenta) and antibodies against the MYC epitope to label Zip3-MYC (green). (**A**) In wild-type strains (AM4171), structured illumination microscopy reveals Zip3-MYC foci directly in between lengthwise-aligned homologous chromosome axes in mid-meiotic prophase nuclei, reflecting the embedded distribution of Zip3 complexes within the central region of SCs. (**B**) The position of Zip3-MYC foci in between lengthwise-aligned axes is unchanged but the number of robust Zip3-MYC foci is severely diminished in *zip1[Δ2–9]* strains (AM4277). Zip3-MYC’s location within the central element substructure of the SC in this strain is also indicated in this image, where Zip3-MYC (green) is co-labeled with antibodies that target Ecm11-Gmc2 (magenta). Scale bar, 1 μm.

**Fig 13:**
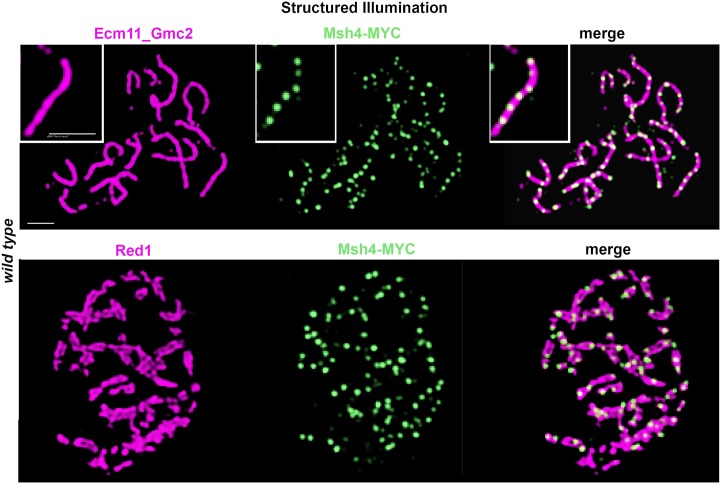
Msh4-MYC localizes to the SC central element sub-structure. Images show representative surface-spread meiotic nuclei from a wild-type strain (K1268) carrying a MYC-tagged *MSH4* gene. The surface-spread nuclei are labeled with antibodies against Ecm11-Gmc2 central element proteins (magenta, top row), or the chromosomal axis protein Red1 (magenta, bottom row), in conjunction with antibodies against the MYC epitope (green). The increased resolving power of structured illumination microscopy reveals that Msh4-MYC foci directly embed within the central element substructure of the SC (see zoomed inset in top row). Scale bar, 1 μm.

As expected based on ultrastructural images of recombination nodules along the length of synapsed chromosomes [20, 22, 52], we found that meiotic recombination proteins embed within the SC central region in budding yeast. Structured illumination microscopy (SIM) in conjunction with antibodies targeting the meiotic axis protein Red1 and the central element protein(s) Ecm11 or Gmc2 reveal MutSγ and Zip3 foci at the midline of the SC, embedded within the SC central element. Singular foci of epitope-tagged MutSγ protein Msh4-MYC and Zip3-MYC localize directly between aligned Red1-labeled axes, where the SC central element substructure is positioned (Figs 12 and 13). When antibodies targeting SC central element proteins are used to label the SC central element directly, Msh4 foci are observed embedded directly in the linear Ecm11-Gmc2 structures at the midline of the SC (Fig 13).

**Fig 14:**
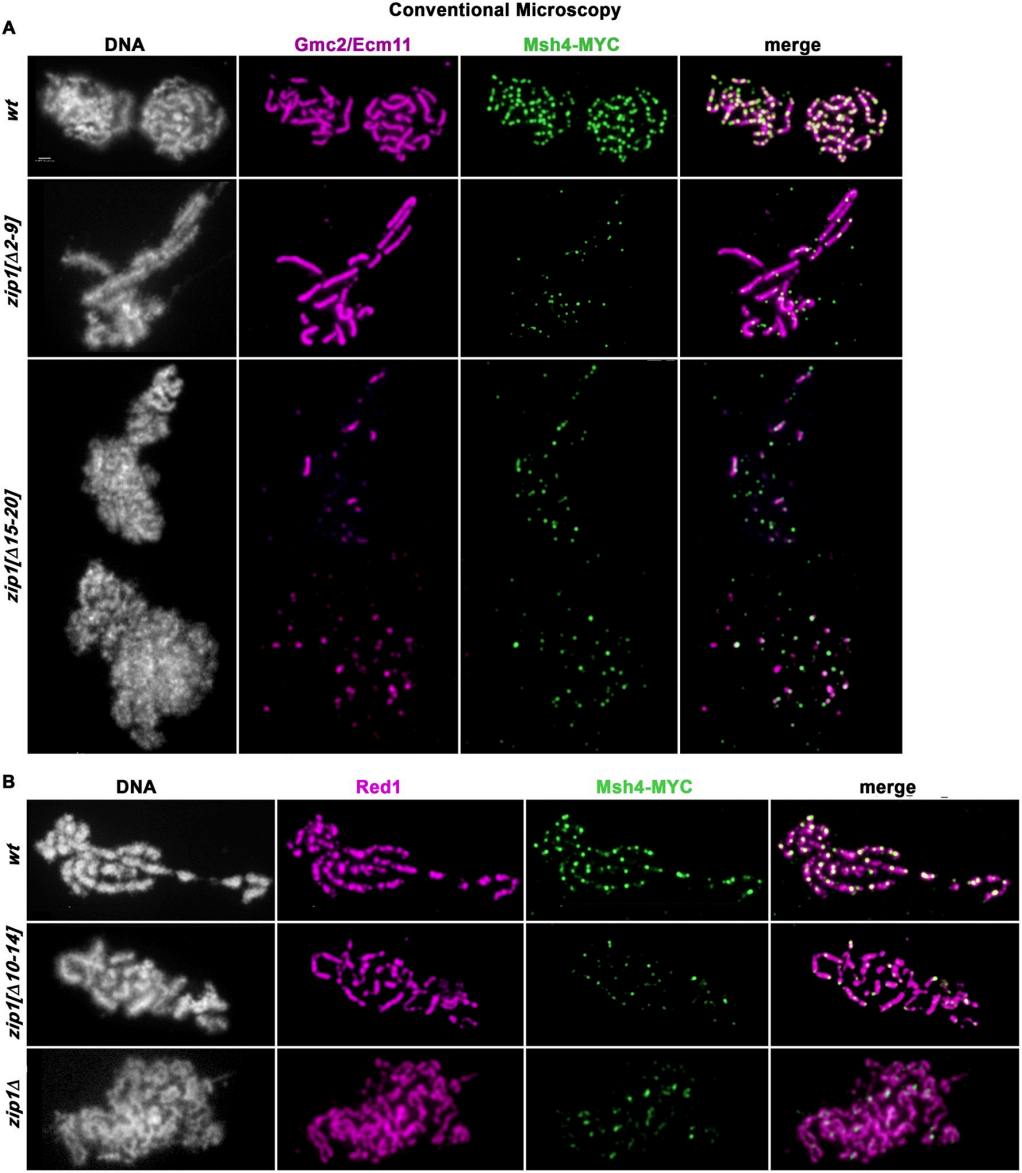
Msh4-MYC foci are reduced in number and diminished in size in *zip1[Δ2–9]* and *zip1[Δ10–14]* strains. Images show representative surface-spread meiotic nuclei labeled with antibodies that target Ecm11-Gmc2 (magenta, top rows) or antibodies against the chromosomal axis protein Red1 (magenta, bottom rows) in conjunction with antibodies that target the MYC epitope to label Msh4-MYC (green). (**A**) In wild-type strains (AM4278; top row), conventional fluorescence microscopy reveals Msh4-MYC foci directly embedded within the SC central element substructure, consistent with the position of Zip3 complexes (which colocalize with Msh4 [28]) within the central region of SCs as shown in Fig 12. The position of Msh4-MYC foci within the central region of the SC is unchanged but the number of robust Msh4-MYC foci is severely diminished in *zip1[Δ2–9]* strains (AM4274; middle row) and in *zip1[Δ15–20]* strains (AM4265; bottom panel including two rows). (**B)** The distribution of Msh4-MYC foci with respect to Red1-labeled chromosome axes is shown for wild type (K1268; top row), *zip1[Δ10–14]* strains (AM4270; middle row), as well as *zip1* null strains (AM4263; bottom row). Scale bar, 1 μm.

In SCs assembled by Zip1[Δ2–9] protein, we observe Zip3-MYC foci embedded within the SC central element, but such Zip3-MYC foci appear strongly diminished in both number and intensity relative to Zip3-MYC foci on meiotic chromosomes from *ZIP1* strains (Fig 12). Similarly, using conventional wide-field fluorescence microscopy we observe a diminished number of bright Msh4-MYC foci on aligned mid-meiotic prophase chromosomes in strains expressing *zip1[Δ2–9]*, *zip1[Δ10–14]* and *zip1[Δ15–20]* relative to *ZIP1* strains, mimicking the Msh4-MYC pattern seen in a *zip1* null strain (Fig 14A and 14B). Furthermore, the Msh4-MYC foci observed on synapsed meiotic prophase chromosomes in *zip1[Δ2–9]* strains do not robustly co-localize with other SIC proteins, such as Zip4-HA, relative to the Msh4-MYC foci assembled on synapsed chromosomes in wild-type meiotic nuclei (S3 Fig).

Our ChIP and cytological studies together indicate that the N terminal twenty residues of the budding yeast transverse filament protein, Zip1, are essential for the proper enrichment of pro-crossover protein Zip3 to meiotic centromeres, to DSBs, and to chromosomal axis sites, and for the accumulation of robust Zip3 and MutSγ foci within the central region of the SC.

## Discussion

**Fig 15:**
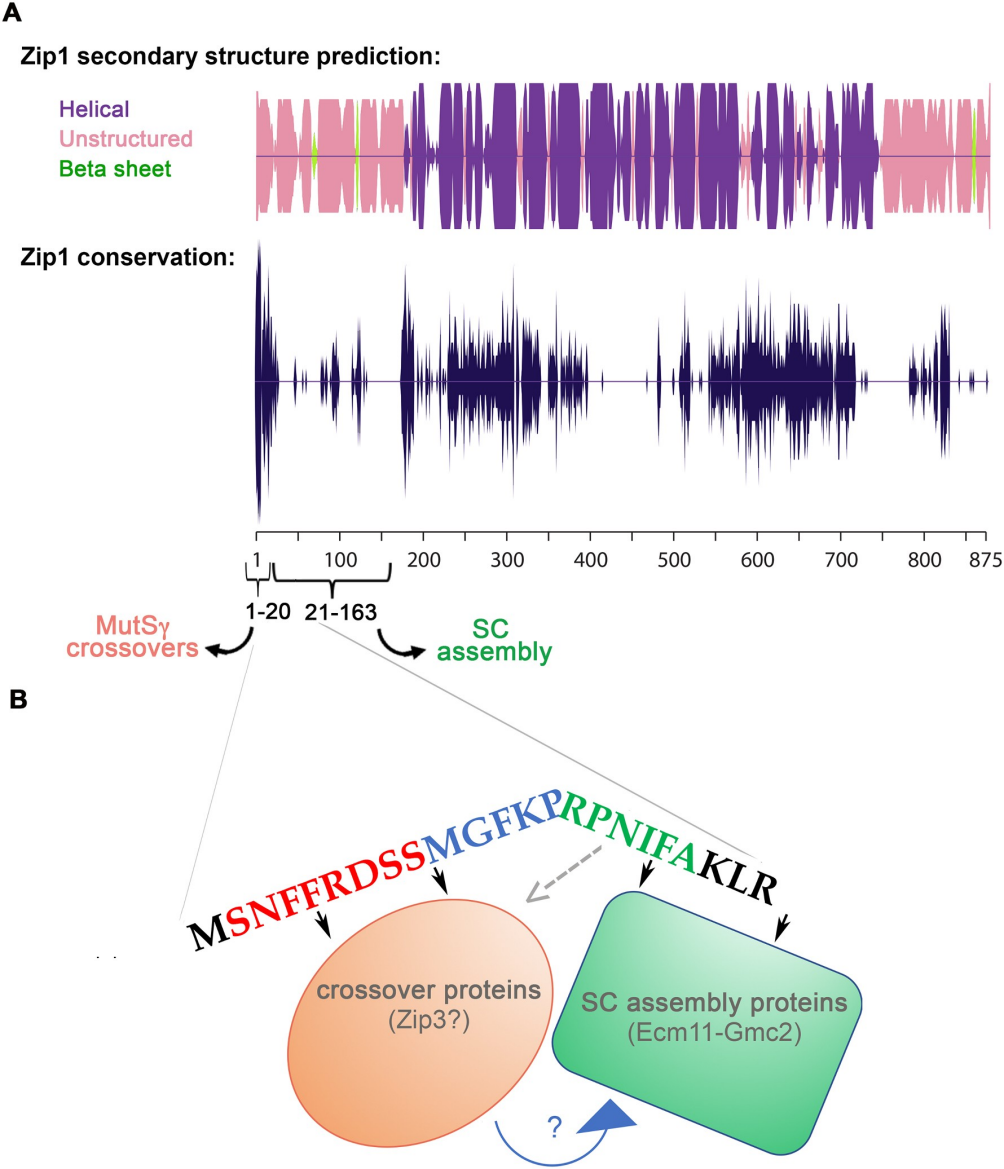
Adjacent conserved regions within Zip1’s N terminus coordinate meiotic crossing over with synapsis: *A model*. **A)** Cartoon illustrates the likelihood of the indicated secondary structure (alpha helical = purple; beta sheet = green; unstructured = pink) across the length of the 875 residue Zip1 protein. Secondary structure prediction was performed using JNet (http://www.compbio.dundee.ac.uk/www-jpred/). The lower line plot indicates the relative conservation of individual amino acid residues across the entire Zip1 protein, using homologs from the following 18 fungal species: *Saccharomyces cerevisiae*, *Candida glabrata*, *Lachancea lanzarotensis*, *Tetrapisispora blattae*, *Tetrapisispora phaffii*, *Kazachstania naganishii*, *Vanderwaltozyma polyspora*, *Torulaspora delbrueckii*, *Zygosaccharomyces bailii*, *Zygosaccharomyces rouxii*, *Kazachstania africana*, *Naumovozyma dairenensis*, *Naumovozyma castellii*, *Kluyveromyces marxianus*, *Kluyveromyces dobzhanskii*. *Eremothecium cymbalariae*, *Ashbya gossypii*, *Ashbya aceri*. Conservation scores are calculated in JalView 2.10 [67] based on multiple sequence alignment (http://www.jalview.org/help/html/calculations/conservation.html). The conservation score is based on the physio-chemical properties of the amino acid residues and is given in arbitrary units from 0–11, where 11 is the most conserved (scores are listed in S6 Table). Stronger conservation is indicated by a longer line above and below the axis. The per-residue Jalview scores were plotted as an area graph in Excel and mirrored in Adobe Illustrator such that the amplitude of the plot signifies conservation: The maximum deviation from the center line indicates a maximum conservation (score = 11), whereas no deviation indicates no conservation (score = 0). Data plotted is listed in S6 Table. The cartoon in **(B)** illustrates the possibility that Zip1’s N terminal 20 residues represent adjacent functionalities: The region corresponding to residues 2–14 is required for MutSγ crossing over, perhaps through a direct interaction with the crossover regulator (and putative E3 SUMO ligase) Zip3, but dispensable for SC assembly *per se*. Our prior characterization of the *zip1[Δ21–163]* internal deletion allele indicates that residues between amino acids 21 and 163 are essential for Zip1’s SC assembly function but completely dispensable for MutSγ crossing over [24]. Data in the current study indicates that residues 15–20 are essential for Zip1’s SC assembly and Ecm11 SUMOylation activity (as demonstrated by the *zip1* null phenocopy displayed by *zip1[Δ15–20]* and *zip1[I18A*, *F19A]* strains). We note that the 15–20 region also plays a role in Zip1’s pro-crossover activity (indicated by gray dotted arrow). We speculate that the adjacency between the functionally distinct regions of Zip1’s N terminus may mechanistically underlie the coordination between MutSγ crossing over and synapsis, by providing a scaffold for direct molecular communication (blue arrow) between crossover factors and synapsis proteins.

SC transverse filament proteins from different organisms have no ancestral relationship but do share a conserved dual functionality: an activity that facilitates interhomolog crossover recombination events between DNA duplexes and a capacity to assemble the tripartite SC structure on meiotic chromosomes. Despite the absence of primary sequence conservation, SC transverse filament proteins from different taxa typically consist of an extended central “core” predicted to assemble coiled-coil, flanked by predicted unstructured N and C terminal regions. Sequence alignment of SC transverse filament proteins from related species reveal highly conserved residues throughout the central helical core of the protein, but also limited clusters of conserved amino acid residues within the N and C predicted unstructured domains (for a mammalian SC transverse filament example, see [53]; Fig 15A illustrates this point for the budding yeast transverse filament, Zip1). These small regions of conservation likely reflect functional residues, perhaps those that form an interaction interface for a partner protein.

In prior work we reported our discovery that a large in-frame deletion of Zip1’s predicted unstructured N terminal region (*zip1[Δ21–163]*; originally created in [54]), encodes a separation-of-function Zip1 protein that fails to assemble mature SC but is completely capable of executing Zip1’s role in MutSγ crossing over [24]. Here we describe two novel non-null *zip1* alleles, *zip1[Δ2–9]* and *zip1[Δ10–14]*, which confer the reciprocal separation-of-function phenotype: robust SC assembly in the absence of MutSγ crossovers. Together with our observation that *zip1[Δ2–163]* and *zip1[Δ2–20]* mutants abolish both SC assembly and MutSγ crossing over, these findings indicate that distinct, adjacent regions within Zip1’s first twenty amino acid residues together serve both of Zip1’s separable functions. Consistent with this possibility, the Zip1’s first twenty residues represent the most highly conserved region across the Zip1 protein among widely divergent yeast species (Fig 15A).

### Adjacent functionally distinct regions within Zip1’s N terminal twenty residues: Interaction domains?

Our analysis of the novel *zip1* alleles reported here leads us to propose that Zip1’s first twenty residues correspond to adjacent interaction domains that directly engage with pro-crossover and pro-synapsis machinery and/or mechanisms, as illustrated in Fig 15B. The absence of Zip1’s residues 2–9 or 10–14 confers a phenotype that strongly resembles the unique phenotype of cells missing the pro-crossover SIC protein, Zip3: *zip1[Δ2–9]*, *zip1[Δ10–14]* and *zip3* strains display some SC assembly despite severely diminished MutSγ crossovers, and hyperSUMOylated forms of the SC central element protein, Ecm11. This constellation of phenotypes is a striking contrast to the synapsis-deficiency and diminished Ecm11 SUMOylation phenotype displayed by mutants that are missing Zip1 altogether or missing other SIC proteins that colocalize with Zip3 at presumed recombination intermediates embedded in the SC, such as Zip2, Zip4, Spo16 or the MutSγ complex [32–34, 37, 38, 55]. The unique, *zip3*-like phenotype of *zip1[Δ2–9]* and *zip1[Δ10–14]* mutants suggests that these mutant meiocytes are missing a specialized capacity to promote Zip3 function.

Zip3 localizes, along with other SIC proteins, to MutSγ foci on mid-meiotic prophase chromosomes in budding yeast and is (along with the other SIC proteins) required for MutSγ -mediated crossover formation [28, 35]. Unlike Zip2, Zip4 and Spo16, however, Zip3 has been implicated in preventing unwarranted (recombination-independent) SC formation [45] and is not required *per se* for SC assembly [28]. Zip4 directly interacts with a Zip2-Spo16 complex that is capable of binding branched DNA structures, and can interact with the meiotic axis component Red1, the MutSγ factor Msh5, as well as Zip3 [37]. One possibility is that while Zip2, Zip4 and Spo16 are absolutely critical at recombination sites to establish a suitable foundation on which to initiate SC assembly, Zip3 may be specifically involved in the licensing of SC assembly at certain recombination sites.

Interestingly, we found evidence in support of a specific regulatory role for Zip3 in meiotic recombination as well. Our data indicates that crossover levels in *zip3*, *zip3 msh4*, or *zip1[2–20] msh4* mutants are elevated over the *msh4* single mutant (Table 2, S2 Table) while crossovers in *zip1* meiotic cells resemble the level observed in *msh4* and *zip1 msh4* strains (Table 1). This observation suggests that Zip3 acts not only to promote MutSγ crossing over, but also to ensure that Zip1-associated recombination intermediates are processed in an MutSγ-dependent manner. Under this model, recombination intermediates associated with both Zip3 and Zip1 fail to resolve into interhomolog crossover events when Msh4 is absent, but when Zip3 or both Msh4 and Zip3 are absent, at least some Zip1-associated recombination intermediates can resolve into interhomolog crossovers (through a MutSγ-independent pathway). This model is supported by sequence signatures observed at interhomolog recombination events in *msh4* versus *zip3* mutants [31], which suggest that unbiased resolution of joint molecule recombination intermediates—a type of resolution associated with MutSγ-independent pathways [5, 56]—occurs to a greater extent in *zip3* relative to *msh4* mutants.

Zip3 is a RING domain protein that has been shown to have SUMO ligase activity *in vitro* [46], and to be responsible for the SUMOylation of a fraction of Red1, a meiosis-specific axis associated protein that is required for normal recombination and SC assembly [57–59]. On the other hand, Zip3 normally *prevents* hyperSUMOylation of the SC central element protein, Ecm11. Thus Zip3 likely impacts meiotic recombination and SC assembly mechanisms through both negatively and positively regulating the SUMOylation of several distinct target proteins.

Consistent with a particular role for Zip3 in preventing unwarranted SC assembly via an interaction with Zip1’s N terminal residues, and also with the idea that this Zip1-Zip3 interaction might be required to “trigger” synapsis initiation at MutSγ crossover sites, *zip1[Δ2–9]* and *zip1[Δ10–14]* mutants display an absence of MutSγ crossovers, a failure of stable Zip3 recruitment to several DSB sites, and premature SC assembly (albeit still between homologs). These phenotypes are consistent with the idea that, through an interaction with Zip1’s N terminus, Zip3 not only constrains Zip1-associated recombination intermediates to be MutSγ-dependent (possibly through the SUMOylation of targets such as the Red1 axis-associated protein), but also negatively regulates SC assembly until the successful completion of a specific intermediate event associated with the recombination process. Perhaps negative regulation of Ecm11 SUMOylation is linked to Zip3’s switch-like function in ensuring that SC assembly occurs “in the right place at the right time” (i.e. at a specific type of recombination intermediate, such as one designated to become a MutSγ crossover event). We note, however, that SC assembly is more robust and non-centromeric synapsis initiation events are more abundant in *zip1[Δ2–9]* and *zip1[Δ10–14]* relative to *zip3* mutant strains; this difference could reflect additional pro-SC assembly activities of Zip3, carried out in a manner that is independent of Zip3’s engagement with Zip1’s N terminal tip.

While multiple attempts at two-hybrid and pull-down experiments have failed to reveal evidence of a strong physical interaction between Zip3 and Zip1’s N terminus, cytological support for this interaction comes from the localization of Zip3 at polycomplex structures, aggregates of Zip1 and other SC-associated proteins that form when SC assembly is compromised. While many if not all SIC proteins have been found to localize to Zip1 polycomplex structures, Zip3’s localization shows a greater degree of coincidence with Zip1 throughout the bulk of the polycomplex, for example relative to Zip4 ([34]; Fig 10A) or the MutSγ component, Msh4 [60]. Importantly, Zip3’s localization throughout the bulk of Zip1 polycomplex is not abolished when Zip4 (which has been found to interact with Zip3 [37]) is absent [34], however Zip3’s localization to the bulk of Zip1 polycomplex is completely abolished by the loss of Zip1’s residues 2–9 or 10–14 (Fig 10A). Finally, Zip1[Δ2–9] and Zip1[Δ10–14] have lost Zip1’s capacity to recruit Zip3 to sites of recombination initiation (Fig 11). Given our inability to observe a stable interaction between Zip1 and Zip3 via pull downs or two hybrid methods, it seems likely that Zip3 interfaces with Zip1 in a manner that is dependent on other proteins, on molecular structures nearby, such as the DNA joint molecule itself, or on a relatively unstable Zip1 structural configuration.

Based on the absence of SC assembly in meiotic cells expressing *zip1[Δ15–20]*, *zip1[15–20→A]* and *zip1[I18A*, *I19A]*, we furthermore conclude that Zip1’s first twenty residues correspond to (perhaps in an overlapping manner) at least one interaction domain for an SC assembly factor or complex of factors. Unlike the limited nature of the region within Zip1’s N terminal 163 amino acids that is required for MutSγ crossovers (twenty residues, based on the fact that *zip1[Δ21–163]* is fully capable of MutSγ crossing over [24]), groups of residues that are critical for allowing Zip1 to assemble SC may be distributed throughout the entire N terminal region encompassed by residues 15–163, as both *zip1[Δ15–20]* and *zip1[Δ21–163]* fail to assemble SC. Nevertheless, we propose that the region overlapping residues 15–20 interfaces with components serving an SC assembly function, based on the fact that alteration of two adjacent residues (I18 and I19) at Zip1’s extreme N terminus (a change that is unlikely to alter the overall length or structure of the rod-like protein) completely abolishes Zip1’s capacity to build the SC.

We previously demonstrated that *zip1[Δ21–163]* phenocopies the *ecm11* and *gmc2* null mutant phenotype (a failure in SC assembly but proficiency in crossing over), suggesting that this N terminal region of Zip1 functionally interacts with the central element in order to assemble SC [24]. Moreover, Leung et. al (2015) demonstrated that Zip1’s N terminal 346 residues is sufficient to promote Ecm11 SUMOylation in vegetative (non-meiotic) cells, provided that Gmc2 is also expressed. These data suggest that the N terminal region of Zip1 is able to engage with the Ecm11-Gmc2 proteins, perhaps in a direct manner or perhaps indirectly through a protein expressed in both meiotic as well as mitotic cells [61]. Similar to the uncertainty about whether Zip3 interacts with Zip1 in a direct manner, apart from the genetic interactions found for Zip1 and Ecm11 and the coincidence of Ecm11 and Gmc2 at the midline of SC (where Zip1 N termini also reside [13]), strong evidence of a direct physical interaction between Zip1 and Ecm11 or Gmc2 does not yet exist.

### The adjacency between Zip1’s pro-crossover and a pro-synapsis regions may serve as a liaison to coordinate SC assembly with intermediate steps in recombination

Finally, we note the tantalizing possibility that the *adjacency* between the putative pro-crossover and pro-synapsis regions of Zip1’s N terminus is functionally important for ensuring that SC assembly occurs in coordination with intermediate steps in the MutSγ crossover recombination pathway. Specifically, we speculate that Zip1 may physically connect crossover recombination events to SC assembly through a mechanism that is based, at least in part, on its capacity to stabilize Zip3 at its N terminus. Here, Zip3 would be expected to be in close proximity to putative pro-synapsis factors stabilized (perhaps in conjunction with other SIC proteins) by the adjacent region in Zip1, and thus could potentially be oriented appropriately to regulate the extent of SUMOylation of SC central element protein Ecm11.

### SCs assembled in the absence of MutSγ crossing over in budding yeast may be less stable

During the course of analyzing SC assembly over a time course of meiotic progression in our mutants we found that SCs assembled in *zip3*, *zip1[D2-9]* and *zip1[10–14]* strains (corresponding to SCs assembled in the absence of MutSγ crossovers) assemble earlier than wild-type SC structures, and appear to be less capable of persisting during an *ndt80*-mediated, meiotic prophase arrest (Fig 4, S2 Fig). Pattabiraman (2017) found that a MutSγ-associated process affects the dynamic properties of *C*. *elegans* SC [39]; our set of preliminary observations raises the intriguing possibility that the MutSγ crossover pathway influences the structure and/or dynamics of budding yeast SCs in a similar fashion.

## Methods

### Strains

Yeast strains used in this study are isogenic to BR1919-8B [62] and were created using standard genetic crosses and manipulation procedures. CRISPR-Cas9 methodology was utilized to create unmarked alleles (described below). Strains for crossover analysis carry an *hphMX4* cassette inserted near the chromosome III centromere, *ADE2* inserted upstream of the *RAD18* locus, a *natMX4* cassette inserted near the *HMR* locus, *TRP1MX4* inserted 62 bp downstream of the *SPO11* locus (Kee and Keeney, 2002), *URA3* replacing *SPO13*, and *LYS2* inserted on chromosome VIII at coordinate 210,400 bp. Strains SYC107, SYC149, SYC151 and K914 do not carry *THR1* nor *LYS2* on chromosome VIII. Zip3 and Zip4 epitope tags (MYC and HA, respectively) are positioned internal to the gene ORFs, as described in [34]. ChIP and qPCR experiments were performed in strains of the SK1 genetic background (S4 Table).

### Creation of unmarked *zip1* alleles using CRISPR-Cas9

Custom *zip1* alleles were created in two sequential transformation steps: The first step is the creation of a “base strain”, which entails replacing the DNA sequences to be altered (in this case, within the *ZIP1* locus) with the *kanMX4* dominant drug resistance cassette. Next, ~500 ng of a CRISPR-Cas plasmid *pRS425-Cas9-kanMX*, created by Gang Zhao in Bruce Futcher’s laboratory (Stony Brook University), is transformed into the *zip1-kanMX4* base strain along with custom DNA sequences (“healing fragments”) that carry the desired alteration in DNA sequence as well as homology to sequences flanking the *kanMX4* insert. *pRS425-Cas9-kanMX* is a yeast two micron plasmid carrying sequences that encode the *LEU2* gene, the *CAS9* gene (driven by the *TEF1* promoter) and a unique *CRISPR* guide RNA (driven by the RNA polymerase III promoter, *SNR52)* that targets the *kanMX* gene. For our strains, “healing fragments” typically correspond to two overlapping *ZIP1* DNA sequences sharing at least twenty bases of overlap that encode the DNA changes desired in the new allele. These DNA fragments were amplified by PCR using a template with sequences containing the wild type *ZIP1* gene. The 5’ healing fragment is created using a reverse primer containing the desired DNA changes and a forward primer that has homology to *ZIP1* sequences 5’ of the *kanMX4* insertion, whereas the 3’ healing fragment is created using a forward primer containing the desired DNA changes (usually a reverse complement DNA fragment to the reverse primer used to create the 5’ fragment) and a reverse primer corresponding to *ZIP1* sequences 3’ to the *kanMX4* insertion. Primers are positioned such that the PCR products contain have at least 50bp of DNA homologous to the regions flanking *kanMX4* in the base strain. After transformation of the *zip1-kanMX4* strain with *pRS425-Cas9-kanMX* plasmid DNA along with both “healing” DNA fragments, cells are plated onto synthetic medium lacking leucine and incubated for up to 10 days at thirty degrees Celsius. Leu+ colonies survive due to the presence of the *pRS425-Cas9-kanMX* plasmid and because of a DNA repair event that replaced *kanMX4* with “healing fragment” sequences. Leu+ colonies are screened for G418 sensitivity; G418-sensitive transformants are struck out on YPD media to isolate single colonies and then genotyped by PCR. For potential mutant allele transformants (based on the PCR genotyping), the entire gene ORF is sequence verified.

### Cytological analysis and imaging

Meiotic nuclei were surface spread on glass slides and imaged as described in [24]. The following primary antibodies were used: affinity purified rabbit anti-Zip1 (1:100, raised at YenZym Antibodies, LLC, against a C terminal fragment of Zip1, as described in [43], mouse anti-cMYC (1:200, clone 9E10, Abcam). Mouse anti-Gmc2 antibodies were raised against purified Gmc2 protein, and guinea pig anti-Gmc2_Ecm11 antibodies were raised against a co-purified protein complex (ProSci Inc.). These antibodies were used at 1:800. Chicken anti-HA (1:100, Abcam), and rabbit anti-Red1 (1:100, a kind gift from G.S. Roeder, [59]) were also used. Secondary antibodies conjugated with Alexa Fluor dyes were purchased from Jackson ImmunoResearch and used at 1:200 dilution. Microscopy and image processing were performed using a Deltavision RT imaging system (General Electric) adapted to an Olympus (IX71) microscope. Measurements of Zip1, Ecm11, and SC linear structures in Fig 4 and S2 Fig (raw data in S5 Table) were measured manually by K.V.M. *(“ImageK”*), using the measurement tool in the SoftWorx program associated with the Deltavision RT system. Structured illumination microscopy was carried out using Applied Precision’s OMX Blaze Structured Illumination Microscope system at The Rockefeller University’s Bio-Imaging Resource Center.

### Calculations and statistical analysis

Genetic crossover data was compiled and processed using an Excel Linkage Macro program, created by Jonathan Greene (Rhona Borts, pers. comm.) and donated by Eva Hoffmann (University of Copenhagen, Denmark). Crossover values (and their standard errors) were obtained using the Stahl lab online tools (https://elizabethhousworth.com/StahlLabOnlineTools/), with the method of Perkins [63]. Non-mendelian segregation is reported in S3 Table. Recombinant spore values were calculated according to the following: 100(*r/t*), where *r* = the number of colonies carrying a chromosome which is recombinant in the interval and *t*; the total number of colonies assessed. Standard error (S.E.) values for random spore analysis were calculated according to the formula: 100(√(r/t)(1-r/t)/t) [64]. All other statistical analyses were carried out using Graphpad Prism or Graphpad InStat (www.graphpad.com).

### Western blot

Western blotting was performed as described previously [13] with the following modifications: Amersham Protran 0.2μm NC was used as the transfer membrane following the manufacturer’s recommendation; after secondary antibody incubation the membrane was processed with a final wash in 100mM Tris-Cl pH 9.5, 100mM NaCl, 5mM MgCl_2_ to boost the HRP- mediated chemiluminescence using Amersham ECL Prime Western Blotting Detection Reagent. Signals were detected on a Syngene G:Box and measured using Syngene GeneTools software; the areas being assessed for measurements were manually refined in order to ensure all and only appropriate data from each lane is collected.

### Chromatin immunoprecipitation

Meiotic cells were processed as described [65], with the following modifications: Lysis was performed in Lysis buffer plus 1 mM PMSF, 50 μg/mL Aprotinin and 1X Complete Mini EDTA-Free (Roche), using 0.5 mm zirconium/silica beads (Biospec Products, Bartlesville, OK). 2 μg of the mouse monoclonal anti-FLAG antibody M2 (Sigma) and 30 μL Protein G magnetic beads (New England Biolabs) were used. Quantitative PCR was performed from the immunoprecipitated DNA or the whole-cell extract using a 7900HT Fast Real-Time PCR System (Applied Biosystems, Thermo Scientific) and SYBR Green PCR master mix (Applied Biosystems) as described [65]. Results were expressed as % of DNA in the total input present in the immunoprecipitated sample. Primers for *GAT1*, *BUD23*, *ERG1*, Axis and *NFT1* loci have been described [50, 51, 66].

## Supporting information

S1 Fig Residues within Zip1’s 2–20 region are required for SC assembly. Representative surface-spread mid-meiotic prophase nuclei from diploids homozygous for *zip1[Δ2–20]* (K1266; top row), or *zip1[Δ 2–163]* (K1267; bottom rows). All strains also carry the *ndt80* null allele, which allows meiotic cultures to accumulate at mid-late prophase stages when full-length SCs are normally present. Mid-meiotic prophase chromosomes are stained with DAPI to label DNA (white), anti-Zip1 (green), and anti-MYC to label Ecm11 (magenta). The merge between Zip1 and Ecm11 channels is shown in the final column. Scale bar, 1 μm.
(PDF)

S2 Fig Replicate time course analysis of SC assembly in wild-type, *zip1[Δ2–9]*, *zip1[Δ10–14]*, *zip3* strains. Each circle in the scatterplots represents the total length of linear assemblies of coincident Ecm11 and Zip1 detected in surface spread meiotic nuclei of wild-type (blue), *zip1[Δ2–9]* (red), *zip1[Δ10–14]* (green) or *zip3* null (orange) mutant strains at 13, 15, 21 or 26 hours after placement into sporulation medium (time points indicated on the *x* axis). 50 nuclei were examined for each strain at every individual time point. Assemblies of SC proteins were considered to be linear if they measured 0.7 μm or greater in length, although some large or adjacent foci potentially were included in these calculations (see arrowhead in Fig 2 and Fig 2 legend). Bars indicate the mean, and standard error of the mean, respectively. P values—calculated using the Mann-Whitney nonparametric statistical test—report the significance of differences in rank distributions between wild type and each mutant at the earliest (13 hour) timepoint, or between the same mutant at 26 versus 21 hour timepoints. Raw data for S2 Fig plots is provided in S5 Table.
(PDF)

S3 Fig Msh4-MYC foci rarely co-localize with Zip4-HA in *zip1[Δ2–9]* strains. Images display the same surface-spread meiotic nuclei shown in the top two rows of Fig 14. In addition to DNA (white) and Msh4-MYC (magenta), Zip4-HA is labeled in green. In wild-type strains (AM4278; top row), Msh4-MYC foci generally appear coincident to or adjacent to Zip4-HA foci. By contrast, the fewer and less bright Msh4-MYC and Zip4-HA foci that localize to synapsed meiotic prophase chromosomes in *zip1[Δ2–9]* strains (AM4274) rarely appear co-localized. Scale bar, 1 μm.
(PDF)

S1 Table Sporulation efficiency and spore viability of strains used for crossover analysis. Sporulation efficiency reflects the fraction of cells that are 2, 3 or 4-spore asci after 5 days on sporulation plates. The frequency of tetrads containing four, three, two, one, or zero viable spores is shown along with the total spore viability (under “% Spore viability”); n.d. = not determined. Full strain genotypes are listed in S4 Table. An asterisk indicates data that was previously published [24, 35].
(PDF)

S2 Table Map distances in *zip3* and additional *zip1* allele strains. Data display and calculations are as in Table 2.
(PDF)

S3 Table Non-Mendelian segregation (gene conversion events) per locus, measured in 4-spore viable tetrads. Shown are the percentages of non-Mendelian segregation events (3:1/1:3 segregation, top; 4:0/0:4 segregation, below) out of the total tetrads analyzed (second column) in each of the indicated strains. Data is derived from 4-spore viable tetrads with no more than 2 gene conversion (non-2:2) events, although cases where adjacent loci segregate non-2:2 were considered a single conversion event. The sum total percentage of observed non-Mendelian events, and the fold increase relative to wild type, is presented, far right. Strains marked with a single asterisk have a different set of genetic markers on chromosome VIII, relative to the wild-type strain used in this analysis. Strains marked with a double asterisk were previously published [24].
(PDF)

S4 Table Strains used in this study. Strains are of the BR1919-8B background [62] except those used for ChIP studies, which are of the SK1 genetic background.
(PDF)

S5 Table Raw data for plots in Fig 4 and S2 Fig. The workbook contains thirty seven sheets. Each sheet contains the SC measurement data for every meiotic nucleus recorded per strain at each timepoint in the timecourse presented in Fig 4 or S2 Fig. For the first timecourse (sheets 1–20), the number of Zip1, Ecm11 and “SC” (coincident Zip1 and Ecm11) linear structures greater than or equal to 0.7 microns is given in the first several columns, and the length (in microns) of each Zip1, Ecm11 or SC stretch is given (oriented horizontally where each column corresponds to an individual stretch). Sheet 21 contains the polycomplex data that is plotted in Fig 4. For the second timecourse, only assemblies of coincident Ecm11 and Zip1 linear structures greater than or equal to 0.7 microns are tallied.
(XLSX)

S6 Table Raw data for plots in Figs 8, 11 and 15. The workbook contains three sheets. Sheet one contains the signal counts measured for the different forms of Ecm11-MYC (unSUMOylated, monoSUMOylated, polySUMOylated, superSUMOylated) observed in meiotic extracts from three experiments. The numbers correspond to the percentage of signal in a protein band corresponding to a given form (i.e. unSUMOylated) as a fraction of the total signal corresponding to all Ecm11-MYC bands in the lane. These data are plotted in Fig 8B. Sheet two contains the raw data corresponding to ChIP analysis of Zip3 binding at various genomic loci. The data from both replicates are shown, except in the case of the *zip1* null, which was performed once. These data are plotted in Fig 11. Sheet three contains the conservation scores for each residue of Zip1, measured in Jalview [67] by analysis of a multiple sequence alignment involving full length Zip1 molecules from the indicated organisms (see Fig 15 legend). The conservation scores are plotted in Fig 15. As described in the Fig 15 legend, the conservation score is based on the physio-chemical properties of each amino acid residue, with 11 being the highest conservation possible and 0 being the lowest. The score for each residue in the 875 amino acid *S*. *c*. Zip1 protein is given across the top row of this worksheet.
(XLSX)
