# Supplementary figures and images for: Crossover recombination and synapsis are linked by adjacent regions within the N terminus of the Zip1 synaptonemal complex protein
Source: PLoS Genet. 2019 Jun 20;15(6):e1008201. doi: 10.1371/journal.pgen.1008201 (PMC6605668; doi:10.1371/journal.pgen.1008201)

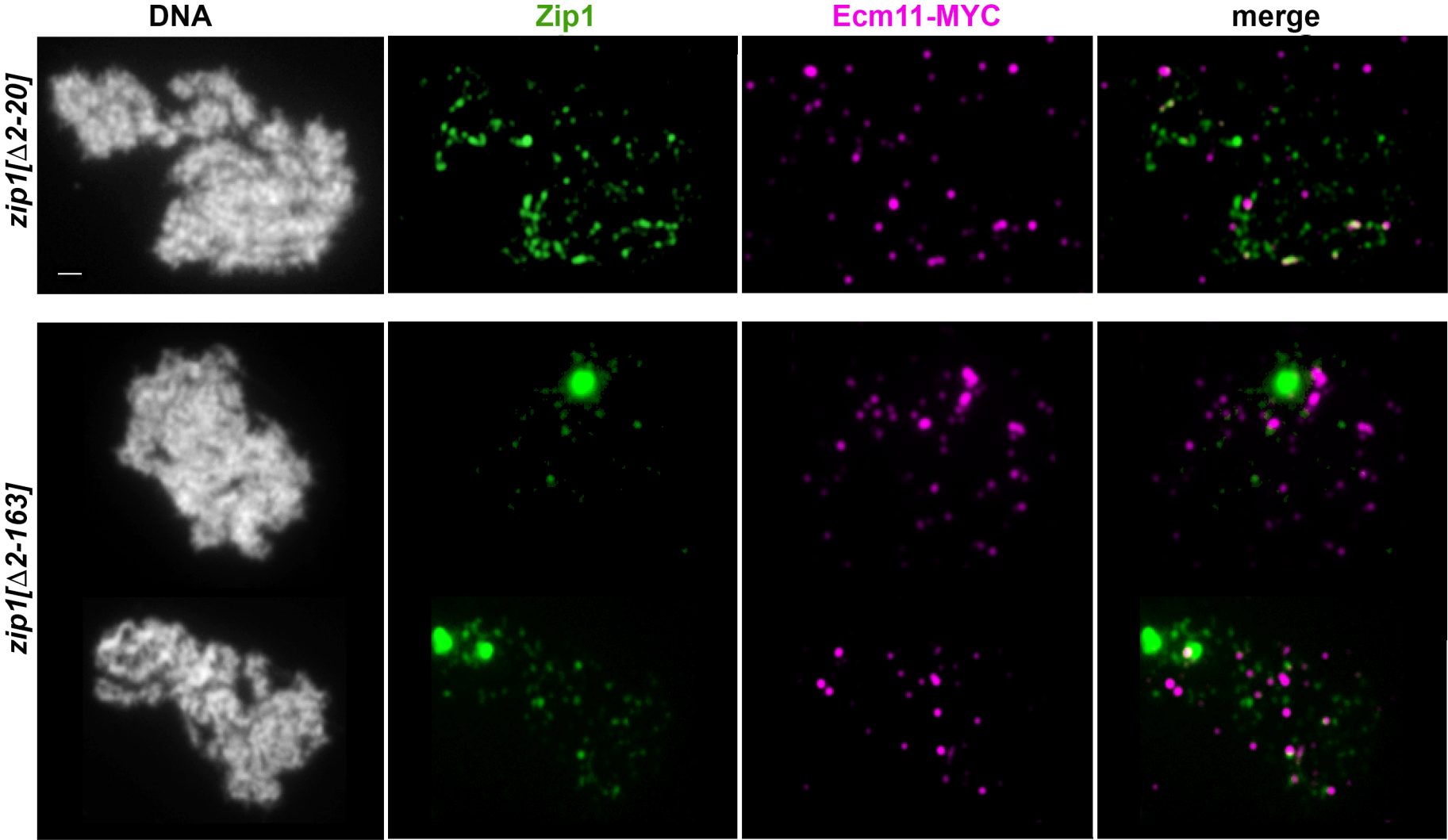

Supplement: S1 Fig — Representative surface-spread mid-meiotic prophase nuclei from diploids homozygous for zip1[Δ2–20] (K1266; top row), or zip1[Δ 2–163] (K1267; bottom rows). All strains also carry the ndt80 null allele, which allows meiotic cultures to accumulate at mid-late prophase stages when full-length SCs are normally present. Mid-meiotic prophase chromosomes are stained with DAPI to label DNA (white), anti-Zip1 (green), and anti-MYC to label Ecm11 (magenta). The merge between Zip1 and Ecm11 channels is shown in the final column. Scale bar, 1 μm. (PDF) [file pgen.1008201.s001.pdf]

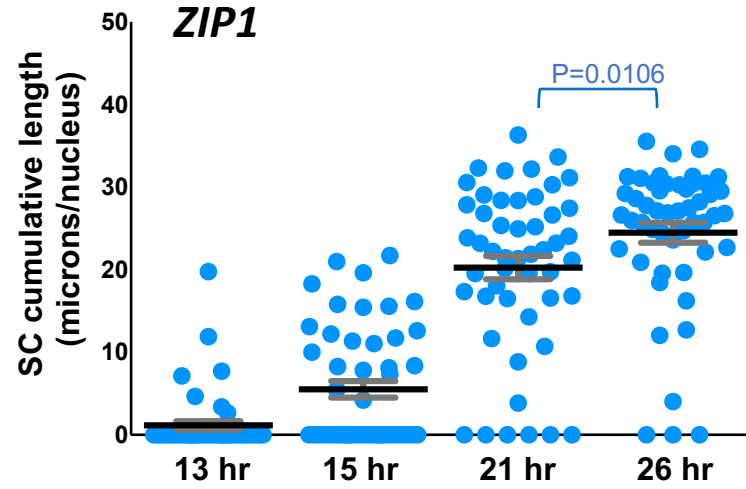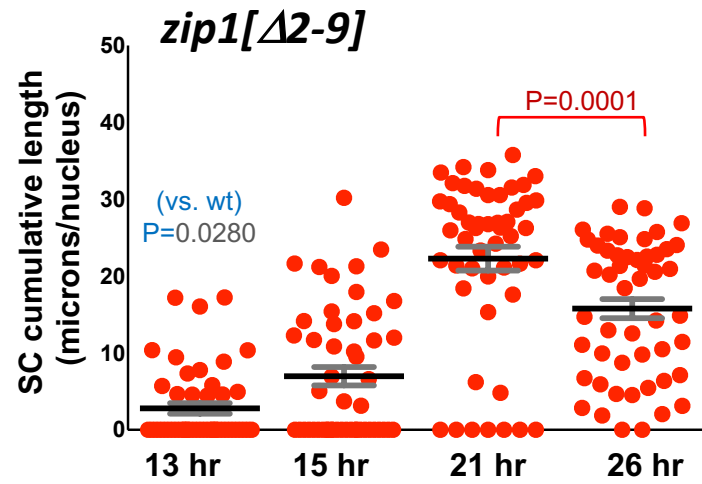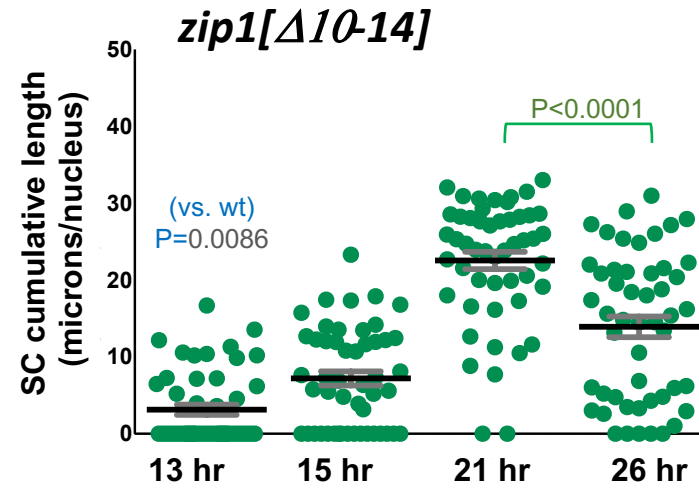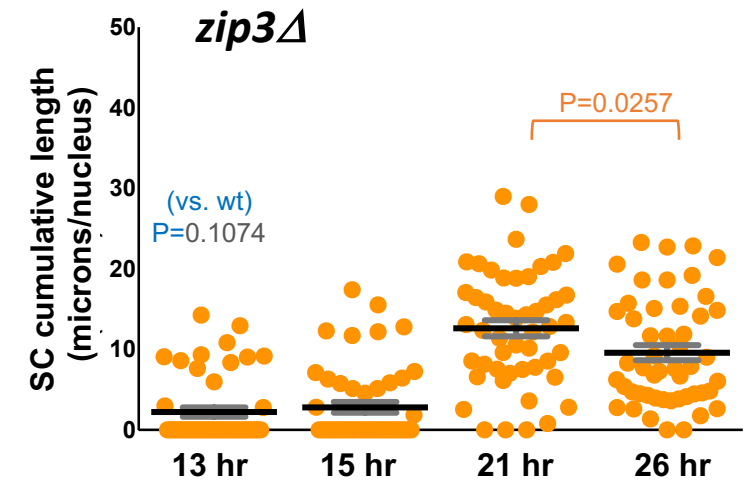

Supplement: S2 Fig — Each circle in the scatterplots represents the total length of linear assemblies of coincident Ecm11 and Zip1 detected in surface spread meiotic nuclei of wild-type (blue), zip1[Δ2–9] (red), zip1[Δ10–14] (green) or zip3 null (orange) mutant strains at 13, 15, 21 or 26 hours after placement into sporulation medium (time points indicated on the x axis). 50 nuclei were examined for each strain at every individual time point. Assemblies of SC proteins were considered to be linear if they measured 0.7 μm or greater in length, although some large or adjacent foci potentially were included in these calculations (see arrowhead in Fig 2 and Fig 2 legend). Bars indicate the mean, and standard error of the mean, respectively. P values—calculated using the Mann-Whitney nonparametric statistical test—report the significance of differences in rank distributions between wild type and each mutant at the earliest (13 hour) timepoint, or between the same mutant at 26 versus 21 hour timepoints. Raw data for S2 Fig plots is provided in S5 Table. (PDF) [file pgen.1008201.s002.pdf]

Conventional Microscopy

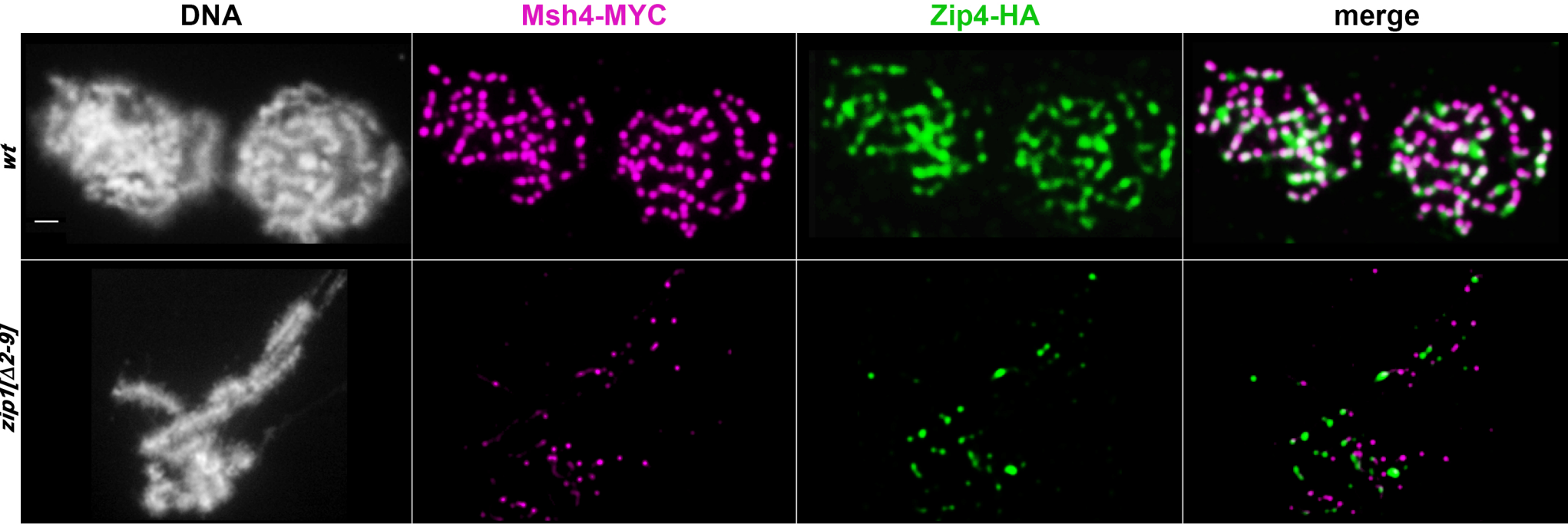

\* nuclei same as those in Fig. 14

Supplement: S3 Fig — Images display the same surface-spread meiotic nuclei shown in the top two rows of Fig 14. In addition to DNA (white) and Msh4-MYC (magenta), Zip4-HA is labeled in green. In wild-type strains (AM4278; top row), Msh4-MYC foci generally appear coincident to or adjacent to Zip4-HA foci. By contrast, the fewer and less bright Msh4-MYC and Zip4-HA foci that localize to synapsed meiotic prophase chromosomes in zip1[Δ2–9] strains (AM4274) rarely appear co-localized. Scale bar, 1 μm. (PDF) [file pgen.1008201.s003.pdf]
