# Supplementary material for: Crossover recombination and synapsis are linked by adjacent regions within the N terminus of the Zip1 synaptonemal complex protein
Source: PLoS Genet. 2019 Jun 20;15(6):e1008201. doi: 10.1371/journal.pgen.1008201 (PMC6605668; doi:10.1371/journal.pgen.1008201)
Supplement: S1 Table — Sporulation efficiency reflects the fraction of cells that are 2, 3 or 4-spore asci after 5 days on sporulation plates. The frequency of tetrads containing four, three, two, one, or zero viable spores is shown along with the total spore viability (under “% Spore viability”); n.d. = not determined. Full strain genotypes are listed in S4 Table. An asterisk indicates data that was previously published [24, 35]. (PDF) [file pgen.1008201.s004.pdf]

Supplementary Table S1. Sporulation Efficiency and Viability; Non-Disjunction frequency

GENOTYPE	(strain)	SPORULATION EFFICIENCY % (n)	# Tetrads	% 4 Spore viable	% 3 Spore viable	% 2 Spore viable	% 1 Spore viable	% 0 Spore viable	% Spore viability	Chromosome III Non-Disjunction (NDJ) %	#NDJ/ # 2 spore viable
pch2Δ	(AM3724)	76 (1100)	757	73	17	8	1	1	**90**	3.3	2/61
pch2Δ msh4Δ	(AM4025)	12 (1031)	826	25	13	23	17	22	**51**	10.1	10/189
pch2Δ zip1Δ	(AM4023)	15 (1023)	828	13	14	21	20	31	**39**	0.6	1/175
pch2Δ zip1Δ msh4Δ	(AM4026)	14 (1037)	453	6	7	22	27	39	**29**	7.1	7/98
pch2Δ zip1[Δ2-163]	(AM3725)	28 (1066)	1098	31	16	22	15	16	**58**	4.2	10/238
zip1[Δ2-163]	(AM3655)	1 (1010)								n.d.	n.d.
zip1Δ *	(CO10)	5 (4503)	80	28	23	20	6	23	**56**	n.d.	n.d.
*WT**	(K842)	57 (1007)	786	92	4	3	0	0	**97**	0.0	0/27
msh4Δ *	(K852)	42 (1005)	1028	42	18	23	15	19	**71**	11.0	26/239
zip1[Δ2-20]	(AM3684)	20 (1061)	1030	60	17	13	4	6	**81**	21.6	29/134
zip1[Δ2-20] msh4Δ	(K1000)	12 (1053)	961	46	16	17	9	12	**69**	20.4	33/162
zip1[Δ2-9]	(MP43)	57 (1011)	852	71	15	8	3	2	**87**	2.9	2/69
zip1[Δ2-9] msh4Δ	(MP46)	36 (1017)	1287	38	17	21	12	12	**64**	4.8	13/269
zip1[Δ10-14]	(SYC107)	54 (3196)	802	79	13	6	1	1	**92**	2.1	1/48
zip1[Δ10-14] msh4	(SYC149)	25 (3000)	750	50	17	15	10	8	**73**	8.6	10/116
zip1[Δ15-20]	(AF8)	51 (1044)	802	72	12	11	2	2	**87**	14.4	13/90
zip1[Δ15-20] msh4Δ	(K914)	34 (1048)	968	57	15	16	6	6	**78**	18.4	29/158
zip1[Δ21-163]	(AF6)	42 (1107)	1025	60	22	12	4	1	**84**	0.8	1/128
*zip1[Δ21-163] msh4Δ**	(SYC151)	41 (1243)	1056	59	11	16	3	11	**76**	8.6	10/116
zip3Δ	(K926)	38 (1067)	866	61	16	14	5	5	**81**	10.0	12/120
zip3Δ msh4Δ	(AM3658/AM3659)	10 (2136)	1049	58	14	18	4	6	**79**	5.4	10/185
zip3Δ zip1[Δ2-9]	(MP52)	49 (1014)	1089	51	15	18	8	8	**73**	8.7	17/196
zip1[N3A,R6A,D7A]	(K1281)	61 (1134)	660	81	12	6	1	1	**93**	5.4	2/37
zip1[F4A,F5A]	(K1309)	54 (1224)	624	69	18	9	2	3	**87**	3.4	2/59
zip1[F4A,F5A] msh4Δ	(K1321)	37 (1284)	264	50	18	20	7	5	**75**	5.8	3/52
zip1[I18A,F19A]	(K1282)	54 (1235)	268	81	9	4	1	3	**91**	8.3	1/12
zip1[I18A, F19A] msh4Δ	(K1328)	47 (1105)	271	52	12	20	7	8	**73**	5.5	3/55

* indicates data that was previously published (Voelkel-Meiman, Johnston et al. 2015, Voelkel-Meiman 2016)
