# Supplementary material for: Crossover recombination and synapsis are linked by adjacent regions within the N terminus of the Zip1 synaptonemal complex protein
Source: PLoS Genet. 2019 Jun 20;15(6):e1008201. doi: 10.1371/journal.pgen.1008201 (PMC6605668; doi:10.1371/journal.pgen.1008201)
Supplement: S2 Table — Data display and calculations are as in Table 2. (PDF) [file pgen.1008201.s005.pdf]

Supplemental Table S2 Genetic map distances in various mutant strains

GENOTYPE (STRAIN)	INTERVAL (CHROMOSOME)	PD	TT	NPD	TOTAL	cM (± SE)	%WT	cM by chrm	%WT by chrm	NPDobs/NPDexp (± SE)
zip1[N3A,R6A,D7A] (K1281)	*HIS4-CEN3* (III)	304	219	3	526	**22.5 (1.4)**	**84**	83.1 (III)	**78**	0.18 (0.11)
	CEN3-MAT (III)	367	156	1	524	**15.5 (1.1)**	**77**			0.14 (0.14)
	MAT-RAD18 (III)	264	255	5	524	**27.2 (1.6)**	**75**			0.20 (0.09)
	RAD18-HMR (III)	354	171	3	528	**17.9 (1.4)**	**78**			0.33 (0.19)
	SPO11-SPO13 (VIII)	289	223	5	517	**24.5 (1.6)**	**63**	51.8 (VIII)	**68**	0.28 (0.13)
	SPO13-THR1 (VIII)	462	52	0	514	**5.1 (0.7)**	**67**			n.d.
	THR1-LYS2 (VIII)	292	224	1	517	**22.2 (1.2)**	**75**			0.06 (0.06)
zip1[F4A,F5A] (K1309)	*HIS4-CEN3* (III)	297	114	3	414	**15.9 (1.6)**	**60**	72.5 (III)	**68**	0.61 (0.36)
	CEN3-MAT (III)	300	113	2	415	**15.1 (1.5)**	**75**			0.42 (0.30)
	MAT-RAD18 (III)	259	148	6	413	**22.3 (2.0)**	**61**			0.67 (0.28)
	RAD18-HMR (III)	272	142	3	417	**19.2 (1.7)**	**84**			0.37 (0.22)
	SPO11-SPO13 (VIII)	326	86	1	413	**11.1 (1.2)**	**28**	29.2 (VIII)	**38**	0.38 (0.38)
	SPO13-THR1 (VIII)	373	34	0	407	**4.2 (0.7)**	**55**			n.d.
	THR1-LYS2 (VIII)	303	101	2	406	**13.9 (1.5)**	**47**			0.52 (0.37)
zip1[F4A,F5A] msh4Δ (K1321)	*HIS4-CEN3* (III)	99	26	2	127	**15.0 (3.7)**	**56**	69.0 (III)	**65**	n.d.
	CEN3-MAT (III)	99	30	0	129	**11.6 (1.9)**	**58**			n.d.
	MAT-RAD18 (III)	82	45	1	128	**19.9 (3.0)**	**55**			n.d.
	RAD18-HMR (III)	86	40	3	129	**22.5 (4.3)**	**98**			n.d.
	SPO11-SPO13 (VIII)	86	42	1	129	**18.6 (3.0)**	**47**	39.9 (VIII)	**52**	n.d.
	SPO13-THR1 (VIII)	115	9	0	124	**3.6 (1.2)**	**47**			n.d.
	THR1-LYS2 (VIII)	90	32	2	124	**17.7 (3.8)**	**60**			n.d.
zip1[I18A, F19A] (K1282)	*HIS4-CEN3* (III)	154	52	0	206	**12.6 (1.5)**	**47**	79.8 (III)	**75**	n.d.
	CEN3-MAT (III)	151	61	0	212	**14.4 (1.6)**	**72**			n.d.
	MAT-RAD18 (III)	119	83	8	210	**31.2 (4.1)**	**86**			1.37 (0.52)
	RAD18-HMR (III)	137	75	3	215	**21.6 (2.8)**	**94**			0.68 (0.40)
	SPO11-SPO13 (VIII)	147	60	1	208	**15.9 (2.1)**	**41**	49.1 (VIII)	**64**	0.37 (0.37)
	SPO13-THR1 (VIII)	171	29	1	201	**8.7 (1.9)**	**114**			1.72 (1.74)
	THR1-LYS2 (VIII)	107	92	1	200	**24.5 (2.2)**	**83**			0.12 (0.12)
zip1[I18A, F19A] msh4Δ (K1328)	*HIS4-CEN3* (III)	117	19	2	138	**11.2 (3.3)**	**38**	64.5 (III)	**61**	n.d.
	CEN3-MAT (III)	111	29	0	140	**10.4 (1.7)**	**52**			n.d.
	MAT-RAD18 (III)	80	52	5	137	**29.9 (4.9)**	**82**			n.d.
	RAD18-HMR (III)	102	36	0	138	**13.0 (1.9)**	**57**			n.d.
	SPO11-SPO13 (VIII)	92	41	1	134	**17.5 (2.9)**	**45**	40.7 (VIII)	**53**	n.d.
	SPO13-THR1 (VIII)	121	7	0	128	**2.7 (1.0)**	**36**			n.d.
	THR1-LYS2 (VIII)	91	35	3	129	**20.5 (4.3)**	**69**			n.d.
zip3Δ (K926)	*HIS4-CEN3* (III)	322	161	10	493	**22.4 (2.1)**	**84**	86.5 (III)	**82**	1.16 (0.38)
	CEN3-MAT (III)	327	167	5	499	**19.7 (1.6)**	**98**			0.54 (0.25)
	MAT-RAD18 (III)	318	153	9	480	**21.6 (2.1)**	**60**			1.13 (0.39)
	RAD18-HMR (III)	306	174	8	488	**22.8 (2.0)**	**100**			0.76 (0.28)
	SPO11-SPO13 (VIII)	335	119	7	461	**17.5 (1.9)**	**45**	35.7 (VIII)	**47**	1.49 (0.58)
	SPO13-THR1 (VIII)	427	44	1	472	**5.3 (0.9)**	**70**			1.83 (1.83)
	THR1-LYS2 (VIII)	369	97	4	470	**12.9 (1.5)**	**44**			1.37 (0.69)
zip3Δ msh4Δ (AM3658/AM3659)	*HIS4-CEN3* (III)	351	195	10	556	**22.9 (1.9)**	**86**	93.1 (III)	**88**	0.87 (0.28)
	CEN3-MAT (III)	367	194	8	569	**21.3 (1.7)**	**106**			0.73 (0.26)
	MAT-RAD18 (III)	344	177	12	533	**23.4 (2.1)**	**64**			1.24 (0.37)
	RAD18-HMR (III)	330	210	12	552	**25.5 (2.0)**	**111**			0.86 (0.26)
	SPO11-SPO13 (VIII)	361	179	8	548	**20.7 (1.8)**	**53**	42.4 (VIII)	**56**	0.83 (0.30)
	SPO13-THR1 (VIII)	464	64	1	529	**6.6 (0.9)**	**87**			0.95 (0.95)
	THR1-LYS2 (VIII)	392	129	5	526	**15.1 (1.5)**	**51**			1.04 (0.47)
zip3Δ zip1[Δ2-9] (MP52)	*HIS4-CEN3* (III)	354	155	5	514	**18.0 (1.6)**	**67**	80.6 (III)	**76**	0.78 (0.24)
	CEN3-MAT (III)	355	171	7	533	**20.0 (1.7)**	**100**			0.78 (0.30)
	MAT-RAD18 (III)	345	159	6	510	**19.1 (1.7)**	**53**			0.75 (0.31)
	RAD18-HMR (III)	317	187	9	513	**23.5 (1.9)**	**103**			0.77 (0.27)
	SPO11-SPO13 (VIII)	343	150	6	499	**18.6 (1.7)**	**47**	39.1 (VIII)	**51**	0.83 (0.35)
	SPO13-THR1 (VIII)	438	39	2	479	**5.3 (1.1)**	**70**			4.76 (3.34)
	THR1-LYS2 (VIII)	354	122	4	480	**15.2 (1.6)**	**52**			0.85 (0.43)
