# Supplementary material for: Crossover recombination and synapsis are linked by adjacent regions within the N terminus of the Zip1 synaptonemal complex protein
Source: PLoS Genet. 2019 Jun 20;15(6):e1008201. doi: 10.1371/journal.pgen.1008201 (PMC6605668; doi:10.1371/journal.pgen.1008201)
Supplement: S3 Table — Shown are the percentages of non-Mendelian segregation events (3:1/1:3 segregation, top; 4:0/0:4 segregation, below) out of the total tetrads analyzed (second column) in each of the indicated strains. Data is derived from 4-spore viable tetrads with no more than 2 gene conversion (non-2:2) events, although cases where adjacent loci segregate non-2:2 were considered a single conversion event. The sum total percentage of observed non-Mendelian events, and the fold increase relative to wild type, is presented, far right. Strains marked with a single asterisk have a different set of genetic markers on chromosome VIII, relative to the wild-type strain used in this analysis. Strains marked with a double asterisk were previously published [24]. (PDF) [file pgen.1008201.s006.pdf]

Supplemental Table S3
Non-mendelian segregation events

**% of events that are 3:1/1:3**			**CHROMOSOME III**					**CHROMOSOME VIII**					
		4 spore		*hphMX@*		*ADE2 @*	*natMX@*	*TRP1MX @*	*spo13::*		*LYS2 @*	**Sum 3:1/1:3**	**Mutant/WT**
**Genotype**	**(strain)**	viable tetrads	*HIS4*	*CEN3*	*MAT*	*RAD18*	*HMR*	*SPO11*	*URA3*	*THR1*	*rm VIII* (210k	**% total**	**fold increase**
**Table 1**													
pch2Δ	(AM3724)	543	3.5	0.6	1.7	3.9	1.8	2.4	0.4	9.2	0.9	**24.3**	**3.6**
pch2Δ msh4Δ	(AM4025)	207	3.4	1.9	1.0	1.9	1.0	3.9	0.5	8.7	1.0	**23.2**	**3.4**
pch2Δ zip1Δ	(AM4023)	100	6.0	1.0	1.0	6.0	2.0	2.0	1.0	8.0	2.0	**29.0**	**4.3**
pch2Δ zip1Δ msh4Δ	(AM4026)	25	8.0	4.0	4.0	0.0	0.0	12.0	4.0	8.0	0.0	**40.0**	**5.9**
pch2Δ zip1[Δ2-163]	(AM3725)	329	4.0	1.2	2.1	4.0	1.5	3.3	3.3	11.9	3.0	**34.3**	**5.1**
**Table 2**													
*WT***	(K842)	682	1.0	0.0	0.1	1.0	0.3	1.2	0.3	2.9	0.0	**6.8**	**1.0**
*msh4Δ ***	(K852)	478	1.5	0.2	0.4	2.3	0.2	3.8	0.8	4.4	0.2	**13.8**	**2.0**
zip1[Δ2-20]	(AM3684)	608	5.1	1.5	3.9	8.9	1.3	8.9	2.6	15.5	3.3	**51.0**	**7.5**
zip1[Δ2-20] msh4Δ	(K1000)	443	3.6	0.5	1.1	5.0	1.1	10.2	1.4	12.9	2.0	**37.7**	**5.5**
zip1[Δ2-9]	(MP43)	595	1.8	0.7	1.5	1.2	0.3	3.2	0.5	4.7	0.0	**13.9**	**2.1**
zip1[Δ2-9] msh4Δ	(MP46)	504	1.0	0.0	0.4	1.8	0.4	4.0	0.0	5.2	0.2	**12.9**	**1.9**
zip1[Δ10-14]	(SYC107)	579	2.1	0.5	1.2	1.7	0.9	3.6	0.5	na	na	**10.5**	**2.7***
zip1[Δ10-14] msh4	(SYC149)	362	2.8	0.0	1.9	2.2	0.0	4.1	0.0	na	na	**11.0**	**2.8***
zip1[Δ15-20]	(AF8)	575	4.0	0.0	1.4	3.3	0.5	3.5	0.2	8.3	0.2	**21.4**	**3.1**
zip1[Δ15-20] msh4Δ	(K914)	560	3.0	0.0	1.3	3.6	0.4	6.8	0.4	na	na	**15.4**	**3.9***
zip1[Δ21-163]	(AF6)	605	3.6	0.2	3.5	5.6	1.5	6.8	1.2	10.4	0.5	**33.2**	**4.9**
*zip1[Δ21-163] msh4Δ***	(SYC151)	616	2.6	0.3	1.5	1.8	0.8	6.0	0.3	na	na	**13.3**	**3.4***
**Table S2**													
zip3Δ	(K926)	475	4.6	1.5	3.2	4.8	1.1	10.3	1.3	6.9	1.5	**35.2**	**5.2**
zip3Δ msh4Δ	(AM3658/AM3659)	604	6.8	1.3	4.8	7.8	1.2	7.8	1.8	11.4	1.5	**44.4**	**6.5**
zip3Δ zip1[Δ2-9]	(MP52)	546	4.8	0.7	1.5	4.8	1.1	7.7	0.5	9.9	0.4	**31.4**	**4.6**
zip1[Δ2-163]	(AM3655)	0											
zip1[N3A,R6A,D7A]	(K1281)	531	0.6	0.4	0.9	0.4	0.4	1.7	0.8	2.4	0.2	**7.7**	**1.1**
zip1[F4A,F5A]	(K1309)	425	2.6	0.5	2.1	0.7	0.9	2.6	0.5	3.8	0.7	**14.4**	**2.1**
zip1[F4A,F5A] msh4Δ	(K1321)	132	1.5	1.5	0.8	1.5	0.0	1.5	0.0	6.1	0.0	**12.9**	**1.9**
zip1[I18A,F19A]	(K1282)	217	5.1	0.0	2.3	0.9	0.0	4.1	0.0	7.4	0.5	**20.3**	**3.0**
zip1[I18A, F19A] msh4Δ	(K1328)	142	2.8	0.0	1.4	0.7	0.7	3.5	0.7	7.0	0.0	**16.9**	**2.5**

**% of events that are 4:0/0:4**												**Sum 4:0/0:4**
**Genotype**	**(strain)**											**% total**
pch2Δ	(AM3724)	543	0.0	0.0	0.0	0.0	0.2	0.0	0.0	0.0	0.0	**0.2**
pch2Δ msh4Δ	(AM4025)	207	0.0	0.0	0.0	0.0	0.0	0.5	0.0	0.0	0.5	**1.0**
pch2Δ zip1Δ	(AM4023)	100	0.0	0.0	1.0	0.0	0.0	0.0	0.0	1.0	0.0	**2.0**
pch2Δ zip1Δ msh4Δ	(AM4026)	25	0.0	0.0	0.0	0.0	0.0	0.0	0.0	0.0	4.0	**4.0**
pch2Δ zip1[Δ2-163]	(AM3725)	329	0.0	0.0	0.0	0.0	0.0	0.0	0.3	0.3	0.3	**0.9**
*WT ***	(K842)	682	0.0	0.0	0.0	0.0	0.0	0.0	0.0	0.0	0.0	**0.0**
*msh4Δ***	(K852)	478	0.0	0.0	0.0	0.0	0.0	0.2	0.0	0.0	0.0	**0.2**
zip1[Δ2-20]	(AM3684)	608	0.0	0.0	0.0	0.0	0.0	0.3	0.0	0.2	0.0	**0.5**
zip1[Δ2-20] msh4Δ	(K1000)	443	0.0	0.0	0.5	0.5	0.0	1.1	0.0	0.9	0.5	**3.4**
zip1[Δ2-9]	(MP43)	595	0.0	0.0	0.1	0.1	0.0	0.2	0.0	0.1	0.1	**0.5**
zip1[Δ2-9] msh4Δ	(MP46)	504	0.0	0.0	0.0	0.0	0.0	0.0	0.0	0.2	0.2	**0.4**
zip1[Δ10-14]	(SYC107)	579	1.4	0.7	0.3	0.7	1.2	0.7	0.9	na	na	**5.9**
zip1[Δ10-14] msh4	(SYC149)	362	0.8	0.0	0.0	0.0	0.0	0.6	0.0	na	na	**1.4**
zip1[Δ15-20]	(AF8)	575	0.0	0.0	0.0	0.0	0.0	0.2	0.0	0.0	0.0	**0.2**
zip1[Δ15-20] msh4Δ	(K914)	560	0.0	0.0	0.2	0.0	0.0	0.2	0.0	na	na	**0.4**
zip1[Δ21-163]	(AF6)	605	0.0	0.0	0.2	0.2	0.2	0.2	0.0	0.0	0.0	**0.7**
*zip1[Δ21-163] msh4Δ***	(SYC151)	616	0.2	0.0	0.0	0.0	0.2	0.3	0.0	0.0	0.0	**0.7**
zip3Δ	(K926)	475	0.0	0.0	0.0	0.0	0.0	0.0	0.0	0.0	0.0	**0.0**
zip3Δ msh4Δ	(AM3658/AM3659)	604	0.3	0.0	0.2	0.2	0.2	0.5	0.0	0.3	0.0	**1.7**
zip3Δ zip1[Δ2-9]	(MP52)	546	0.2	0.0	0.0	0.0	0.0	0.0	0.0	0.2	0.0	**0.4**
zip1[Δ2-163]	(AM3655)	0										
zip1[N3A,R6A,D7A]	(K1281)	531	0.0	0.0	0.0	0.0	0.0	0.2	0.0	0.0	0.0	**0.2**
zip1[F4A,F5A]	(K1309)	425	0.0	0.0	0.0	0.0	0.2	0.0	0.0	0.0	0.0	**0.2**
zip1[F4A,F5A] msh4Δ	(K1321)	132	0.8	0.0	0.0	0.8	0.8	0.8	0.0	0.0	0.0	**3.0**
zip1[I18A,F19A]	(K1282)	217	0.0	0.0	0.0	0.0	0.0	0.0	0.0	0.0	0.0	**0.0**
zip1[I18A, F19A] msh4Δ	(K1328)	142	0.0	0.0	0.0	1.4	1.4	0.7	0.7	2.1	0.7	**7.0**

* strains have different set of genetic markers relative to K842.

** Data for these strains was previously published: (Voelkel-Meiman 2016)
