# Supplementary material for: Crossover recombination and synapsis are linked by adjacent regions within the N terminus of the Zip1 synaptonemal complex protein
Source: PLoS Genet. 2019 Jun 20;15(6):e1008201. doi: 10.1371/journal.pgen.1008201 (PMC6605668; doi:10.1371/journal.pgen.1008201)
Supplement: S4 Table — Strains are of the BR1919-8B background [62] except those used for ChIP studies, which are of the SK1 genetic background. (PDF) [file pgen.1008201.s007.pdf]

Table S4. Strains used in this study

	**GENOTYPE**
**YAM1252**	*lys2ΔNhe his4-260,519 leu2-3,112 MAT**α** trp1-289 ura3-1 thr1-4 ade2-1*<br>*lys2ΔNhe his4-260,519 leu2-3,112 MAT**a** trp1-289 ura3-1 thr1-4 ade2-1*
**K842**	*lys2ΔNhe HIS4 leu2-3,112 hphMX4@CEN3 MAT**α** ADE2@RAD18 natMX4@HMR*<br>*lys2ΔNhe his4-260,519 leu2-3,112 CEN3 MAT**a** RAD18 HMR*<br>*trp1-289 ura3-1 TRP1MX4@SPO11 spo13::URA3 THR1 210kb ade2-1*<br>*trp1-289 ura3-1 SPO11 SPO13 thr1-4 LYS2@210kb ade2-1*
**AM3724**	K842 homozygous *pch2::kanMX4*
**AM4025**	K842 homozygous *pch2::kanMX4 msh4::LEU2*
**AM4023**	K842 homozygous *pch2::LEU2 zip1::kanMX4*
**AM3725**	K842 homozygous *pch2::kanMX4 zip1[Δ2-163]*
**AM4026**	K842 homozygous *pch2::kanMX4 zip1::kanMX4 msh4::LEU2*
**K852**	K842 homozygous *msh4::kanMX4*
**AM3684**	K842 homozygous *zip1[Δ2-20]*
**K1000**	K842 homozygous *zip1[Δ2-20] msh4::kanMX4*
**MP43**	K842 homozygous *zip1[Δ2-9]*
**MP46**	K842 homozygous *zip1[Δ2-9] msh4::kanMX4*
**SYC107**	K842 homozygous *zip1[Δ10-14] thr1-4 LEU2@152kb XI 193kb XI*<br>*152 kb XI THR1@193kb XI*
**SYC149**	K842 homozygous *zip1[Δ10-14] msh4::kanMX4 thr1-4 LEU2@152kb XI 193kb XI*<br>*152 kb XI THR1@193kb*
**AF8**	K842 homozygous *zip1[Δ15-20]*
**K914**	K842 homozygous *zip1[Δ15-20] msh4::kanMX4*
**AF6**	K842 homozygous *zip1[Δ21-163]*
**SYC151**	K842 homozygous *zip1[Δ21-163] msh4::kanMX4 thr1-4 LEU2@152kb XI 193kb XI*<br>*152 kb XI THR1@193kb*
**K926**	K842 homozygous *zip3::kanMX4*
**AM3658/ AM3659**	K842 homozygous *zip3::kanMX4 msh4::LEU2*
**MP52**	K842 homozygous *zip3::kanMX4 zip1[Δ2-9]*
**AM3655**	K842 homozygous *zip1[Δ2-163]*
**K1281**	K842 homozygous *zip1[N3A, R6A, D7A]*
**K1309**	K842 homozygous *zip1[F4A, F5A]*
**K1321**	K842 homozygous *zip1[F4A, F5A] msh4::kanMX4*
**K1282**	K842 homozygous *zip1[I18A, F19A]*

**K1328**	K842 homozygous *zip1[I18A, F19A] msh4::kanMX*
**Cytology**	
**K320**	YAM1252 *ECM11-13MYC::kanMX4 ndt80::LEU2*<br>*ECM11 ndt80::LEU2*
**AM4064**	K320 homozygous *zip1[Δ2-9]*
**AM4194**	K320 homozygous *zip1[Δ10-14]*
**AM4069**	K320 homozygous *zip1[10-14 all residues changed to A]*
**SYC79**	K320 homozygous *zip1[Δ15-20]*
**K981**	K320 homozygous *zip1[15-20 all residues changed to A]*
**AM4066**	K320 homozygous *zip3::hphMX4*
**K1266**	K320 homozygous *zip1[Δ2-20]*
**K1267**	K320 homozygous *zip1[Δ2-163]*
**K969**	K320 homozygous *zip1[N3A, R6A, D7A]*
**AM4067**	K320 homozygous *zip1[F4A, F5A]*
**K985**	K320 homozygous *zip1[I18A, F19A]*
**AM4203**	YAM1252 homozygous *CTF19-MYC::kanMX4 ndt80::LEU2*
**CO10**	YAM1252 homozygous *zip1::UAR3*
**AM4204**	AM4203 homozygous *zip3::LYS2*
**AM4231**	AM4203 homozygous *zip1[Δ2-9]*
**AM4175**	AM4203 homozygous *zip1[Δ10-14]*
**AM4174**	YAM1252 *ZIP3-MYC/ZIP3 ZIP4/ZIP4-HA* homozygous *spo11::kanMX4 ndt80::LEU2*
**AM4253**	AM4174 homozygous *zip1[Δ2-9]*
**AM4173**	AM4174 homozygous *zip1[Δ10-14]*
**AM4256**	AM4174 homozygous *zip1[Δ15-20] spo11::hphMX4*
**AM4350**	AM4174 homozygous *zip1[N3A, R6A, D7A] spo11::ADE2*
**AM4340**	AM4174 homozygous *zip1[F4A, F5A] spo11::ADE2*
**AM4343**	AM4174 homozygous *zip1[Δ21-163] spo11::ADE2*
**AM4171**	YAM1252 *ZIP3-MYC/ZIP3 ZIP4/ZIP4-HA* homozygous *ndt80::LEU2*
**AM4277**	AM4171 homozygous *zip1[Δ2-9]*
**K1268**	YAM1252 homozygous *MSH4-13MYC::kanMX4 ndt80::LEU2*

**AM4278**	K1268 *ZIP4/ZIP4-HA*
**AM4274**	K1268 homozygous *zip1[Δ2-9] ZIP4-HA*
**AM4270**	K1268 homozygous *zip1[Δ10-14]*
**AM4265**	K1268 homozygous *zip1[Δ15-20]*
**AM4363**	K1268 homozygous *zip1::URA3*
**AM4458/AM4462**	YAM1252 homozygous *GFP-LacI@URA3 lacO-LEU2@CENIV ndt80::LEU2*
**AM4440/AM4441**	AM4458 homozygous *zip1[Δ2-9]*
**AM4446**	AM4458 homozygous *zip1[Δ10-14]*
**AM4453**	AM4458 homozygous *zip3::hphMX4*
**Western Blot**	
**AM2712**	YAM1252 *ECM11-13MYC::kanMX4 ndt80::LEU2*<br>*ECM11-13MYC::kanMX4 ndt80::LEU2*
**MP39**	AM2712 homozygous *zip1[Δ2-9]*
**SYC96**	AM2712 homozygous *zip1[Δ10-14]*
**SYC97**	AM2712 homozygous *zip1[Δ15-20]*
**AM3719**	AM2712 homozygous *zip3::hphMX4*
**AM3662**	AM2712 homozygous *zip1[F4A, F5A]*
**AM3628**	AM2712 homozygous *zip1[N3A, R6A, D7A]*
**AM3656**	AM2712 homozygous *zip1[P14A, P16A]*
**AM2784**	AM2712 homozygous *zip1::URA3*
**K986**	AM2712 homozygous *zip1[I18A, F19A]*
**IP**	
**ORD9670**	SK1 *lys2 leu2-k MAT**α** ho::LYS2 ura3 arg4-nsp.bgl ZIP3-HIS6-FLAG3::kanMX4*<br>*lys2 leu2-k MAT**a** ho::LYS2 ura3 arg4-nsp.bgl ZIP3-HIS6-FLAG3::kanMX4*
**AM3946 (VBD1872)**	ORD9670 homozygous *zip1[Δ2-9]*
**AM3951 (VBD1873)**	ORD9670 homozygous *zip1[Δ10-14]*
**ORD9689**	ORD9670 homozygous *zip1::kanMX*

